# The role of the polysaccharide capsule as a novel virulence-associated trait of *Stenotrophomonas maltophilia* complex bacteria

**DOI:** 10.1128/mbio.01635-26

**Published:** 2026-09-11

**Authors:** Laurita Klimkaitė, Aleksandras Konovalovas, Jūratė Skerniškytė, Dukas Jurėnas, Asta Mačionienė, Silvija Kiverytė, Julija Armalytė

**Affiliations:** 1 Department of Biochemistry and Molecular Biology, Institute of Biosciences, Life Sciences Center, Vilnius University 54694, Vilnius, Lithuania; 2 Department of Molecular Biology, Faculty of Sciences, Université Libre de Bruxelles 89092 , Gosselies, Belgium; 3 WEL Research Institute 630407 , Wavre, Belgium; 4 Center of Laboratory Medicine, Vilnius University Hospital Santaros Clinics 58939, Vilnius, Lithuania; 5 Department of Physiology, Biochemistry, Microbiology and Laboratory Medicine, Faculty of Medicine, Institute of Biomedical Sciences, Vilnius University 54694, Vilnius, Lithuania; Universiteit Gent, Gent, Belgium

**Keywords:** *Stenotrophomonas maltophilia* complex, polysaccharide capsule, virulence

## Abstract

The polysaccharide capsule is recognized as a major virulence factor of many bacterial pathogens, playing a significant role in evading host immunity. However, it has not yet been characterized for the bacteria within the emerging multidrug-resistant opportunistic pathogens of the *Stenotrophomonas maltophilia* complex. In this study, we aimed to evaluate whether bacteria of the *S. maltophilia* complex form a bona fide polysaccharide capsule and determine its virulence-related functions. We demonstrated that a part of clinical and environmental isolates of the *S. maltophilia* complex forms a polysaccharide capsule and identified a distinct group IV-like, Wzx/Wzy-dependent capsule-associated locus architecture in these bacteria, with capsule-associated genes organized within one or two loci. Comparison of capsulated clinical isolates and their capsule-deficient mutants showed that the polysaccharide capsule protects bacteria of the *S. maltophilia* complex from blood plasma-mediated killing, phagocytosis, and desiccation. However, formation of the polysaccharide capsule can also limit biofilm formation and adhesion to epithelial cells in some isolates, suggesting a potential trade-off for the bacteria. Altogether, our results pinpoint the polysaccharide capsule as one of the previously undescribed virulence-associated factors of the *S. maltophilia* complex, enhancing its ability to escape host innate immunity mechanisms and contributing to pathogenicity.

**IMPORTANCE:**

The emerging pathogens employ a variety of diverse virulence strategies, which must be thoroughly investigated to uncover effective ways of preventing and combating their infections. In this work, emerging multidrug-resistant bacterial pathogens belonging to the *Stenotrophomonas maltophilia* complex were analyzed, with the goal of identifying their mechanisms responsible for evasion of the host immune system. A polysaccharide capsule, a previously uncharacterized virulence-associated trait of *S. maltophilia* complex bacteria, was identified and characterized in this work, revealing both the genetic factors required for its synthesis and its biological importance to the bacteria. This work expands our understanding of the survival strategies of emerging multidrug-resistant bacteria.

## INTRODUCTION

*Stenotrophomonas maltophilia* is an aerobic gram-negative multidrug-resistant opportunistic human pathogen of environmental origin, causing a variety of infections in immunocompromised patients. Although the global spread and pathogenic potential of *S. maltophilia* have been observed only since the late 1990s (1), currently, *S. maltophilia* infections are reported worldwide, with the World Health Organization listing *S. maltophilia* as one of the leading multidrug-resistant pathogens (2, 3). *S. maltophilia* is usually associated with respiratory tract and bloodstream infections, but it can also cause various other infections, such as in the eye, heart, nervous system and spinal cord, gastrointestinal tract, liver, urinary tract, skin, soft tissue, and bones (4). *S. maltophilia* is most notorious for causing chronic lung infections in patients suffering from cystic fibrosis (5) and its ability to form biofilm on various surfaces, particularly catheters (6, 7). Because of a variety of virulence and resistance traits, especially the multidrug resistance phenotype, infections caused by *S. maltophilia* are difficult to treat and are linked to substantial mortality rates ranging from 21% to as high as 69% (8).

Recent genomic studies identified that the previously considered single species of *S. maltophilia* includes multiple cryptic species, impossible to differentiate from other closely related ones in the genus *Stenotrophomonas* using conventional classification methods (3, 9). Due to their high genetic similarity to *S. maltophilia*, 24 species-level clades were grouped into the *S. maltophilia* complex, sharing >99.0% 16S rRNA gene sequence similarity (3, 9). Phylogenetic analysis of sequenced genomes revealed that in the *S. maltophilia* complex, *S. maltophilia* dominates as a species with 42.8% abundance, followed by *Stenotrophomonas sepilia* (13.6%), *Stenotrophomonas geniculata* (9.9%), and *Stenotrophomonas muris* (9.3%) (9). The majority of the cryptic species from the *S. maltophilia* complex originate from clinical sources (3, 9). However, despite *S. maltophilia* complex bacteria having high clinical relevance, the mechanisms of their pathogenesis remain poorly understood.

A polysaccharide capsule is one of the major virulence traits that bacterial pathogens use in order to escape the host immune response (10). Around 50% of all prokaryotes are considered to encode capsule genes (11); however, this ability can vary, depending on bacterial species and even individual strains (12). The polysaccharide capsule is composed of sugar polymers that surround the cell wall, creating a physical barrier that protects the cell from the environment. Polysaccharide capsules are known to play a significant role in the pathogenesis of various bacteria by protecting pathogens from the components of host immunity, such as phagocytosis (13), the complement system (12), and antimicrobial peptides (14). Capsular polysaccharides can mimic host-tissue molecules, thereby creating a non-immunogenic coating and inhibiting the activation of both innate and adaptive immune responses (15). Polysaccharide capsules can also protect from other stresses that pathogens encounter in clinical settings, such as desiccation (16) and antibiotics (17). Besides playing a role as a protective layer, capsular polysaccharides may function as adhesive polymers, influencing bacterial attachment to surfaces and biofilm formation (18), but can conversely also mask surface proteins and hence hinder adhesion (19).

Our previous study indicated a putative capsule-synthesis phenotype of *Stenotrophomonas* strains (20), while recent work by Li et al. identified capsule synthesis-related genes present in *S. maltophilia* complex isolates (9). Although these findings suggest that the *S. maltophilia* complex bacteria have the ability to form a polysaccharide capsule, no detailed analysis has been conducted to clearly characterize this phenotype or elucidate its biological role. In this work, we analyzed a collection of clinical and environmental isolates of the *S. maltophilia* complex and determined that a part of the analyzed isolates forms a bona fide polysaccharide capsule. We identified a distinct group IV-like capsule-associated locus architecture in the *S. maltophilia* complex, with capsule-associated genes organized either in a more contiguous proximal locus or distributed between two proximal and distal chromosomal loci. Comparing the wild-type isolates and their capsule-deficient mutants, we showed that the polysaccharide capsule protects bacteria from blood plasma-mediated killing, phagocytosis, and desiccation but hinders biofilm formation and adhesion to lung epithelial cells. Altogether, these findings indicate that the polysaccharide capsule is an important virulence-associated factor in the *S. maltophilia* complex, facilitating evasion of the host’s innate immune responses. Furthermore, our results suggest that capsule production might present a fitness cost by decreasing the biofilm formation capacity and hindering the adhesion to epithelial cells.

## RESULTS

### *S. maltophilia* complex isolates exhibit a capsule-producing phenotype

**Fig 1:**
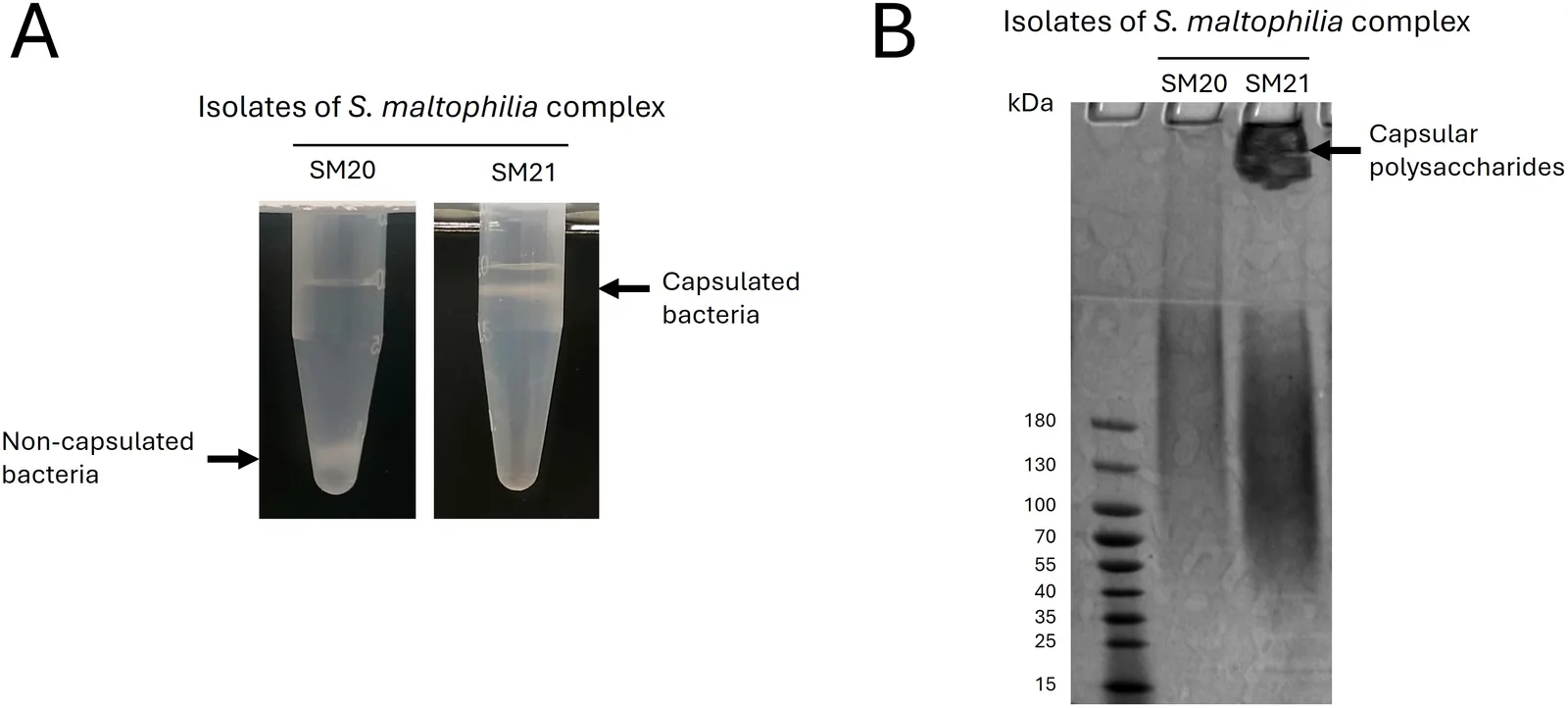
Analysis of polysaccharide capsule phenotype in isolates of *S. maltophilia* complex. Representative images of polysaccharide capsule analysis using a density-dependent gradient test (**A**); fractionation of polysaccharides in SDS-PAGE, followed by Alcian Blue staining (**B**).

Due to the deficiency of research regarding the formation of polysaccharide capsule by the *S. maltophilia* complex bacteria, we aimed to ascertain whether this virulence trait is indeed characteristic of these opportunistic pathogens. To evaluate capsule production, we employed two complementary methods commonly used for polysaccharide capsule detection: a density gradient-based analysis (Fig. 1A) and in-gel staining of bacterial polysaccharides with a saccharide-specific Alcian Blue dye (Fig. 1B). A subset of 42 environmental and 49 clinical *S. maltophilia* complex isolates was analyzed using both approaches, which consistently identified a capsule-producing phenotype in a part of clinical and environmental isolates of the *S. maltophilia* complex (Fig. 1; Fig. S1).

Alcian Blue staining of extracted polysaccharides revealed the presence of high-molecular-weight material that exhibited limited migration through the sodium dodecyl-sulfate polyacrylamide gel electrophoresis (SDS-PAGE) gel and accumulated predominantly within the stacking gel (Fig. 1B; Fig. S1). A similar migration pattern was also reported for capsular polysaccharides of other bacterial species, including *Klebsiella pneumoniae* (21, 22). Density-gradient fractionation demonstrated that isolates producing this high-molecular-weight material exhibited reduced migration through the density gradient (Fig. S1), a characteristic commonly associated with capsule-producing bacteria (23, 24). Based on this consistency, an additional 3 environmental and 56 clinical isolates were screened using density-gradient fractionation alone. Altogether, 42 environmental and 105 clinical isolates were analyzed, revealing a capsule-producing phenotype in 35.7% of environmental isolates and 14.3% of clinical isolates.

### An open pangenome reveals heterogeneous capsule-associated functions across the *S. maltophilia* complex

**Fig 2:**
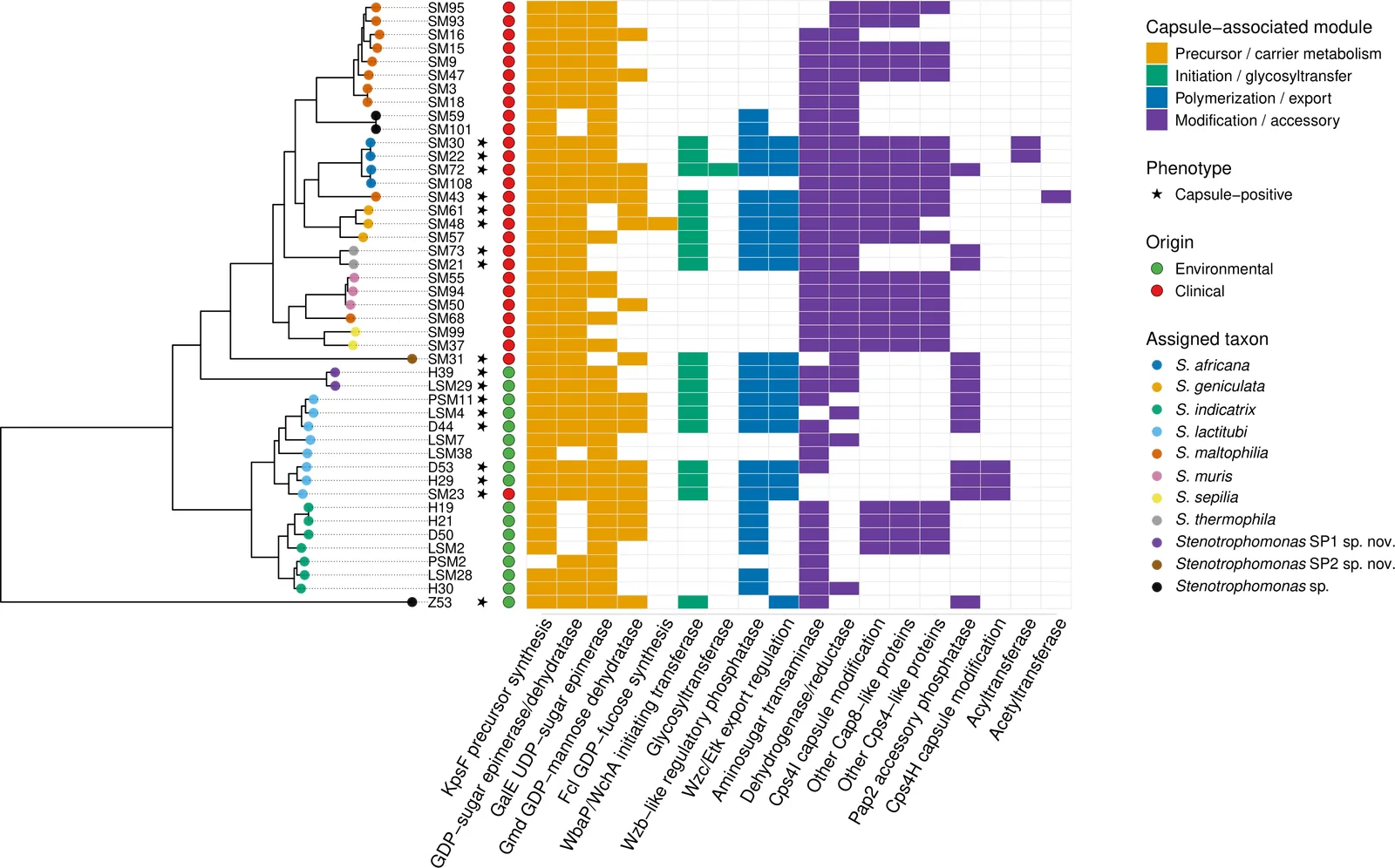
Distribution of genes encoding capsule-associated functional categories across the core-genome phylogeny of *S. maltophilia* complex isolates. The midpoint-rooted core-genome phylogeny includes 45 isolates. Tip-point colors indicate assigned taxon. Black stars mark capsule-positive isolates, and the adjacent origin track indicates an environmental or clinical source. The distribution map shows the presence of detected genes encoding proteins assigned to four capsule-associated functional categories: precursor/carrier metabolism, initiation/glycosyltransferase, polymerization/export, and modification/accessory.

To identify the genes responsible for the expression of the polysaccharide capsule of *S. maltophilia* complex isolates, we performed whole-genome sequencing on a selected set of 45 *S. maltophilia* complex isolates, both phenotypically displaying (*n* = 18) and non-displaying (*n* = 27) polysaccharide capsules. The isolates were randomly selected from both clinical and environmental origin isolate groups. Phylogenetic analysis based on a concatenated alignment of 2,679 single-copy core genes yielded a maximum-likelihood tree, which was midpoint-rooted for downstream visualization and comparative analysis (Fig. 2).

Pangenome analysis revealed a highly open genomic architecture. The cloud genome comprised approximately 11,883 genes, representing around 70% of all orthologous groups, and greatly exceeded both the shell genome, with 2,487 genes, and the core genome, with 2,679 genes. This extensive accessory genome reflects the significant genomic plasticity typical of environmental opportunists (25), and the core-genome phylogeny showed clear structuring by isolate origin, with most clinical and environmental isolates occupying different regions of the tree.

To provide species-level context, each genome was compared by ANI against a *Stenotrophomonas* reference set annotated according to the taxonomic designations proposed by Chauviat et al. (26). These closest-reference assignments placed the isolates across multiple named and proposed lineages within the broader *S. maltophilia* complex, rather than within *S. maltophilia sensu stricto* alone (Fig. 2; Table S1). Clinical isolates were assigned to *S. maltophilia*, *Stenotrophomonas africana*, *S. geniculata*, *S. muris*, *S. sepilia*, *Stenotrophomonas thermophila*, and proposed SP1/SP2 lineages, whereas environmental isolates were mainly represented among *Stenotrophomonas lactitubi* and *Stenotrophomonas indicatrix*. Isolates that did not reach the species-level ANI threshold were conservatively reported as unresolved *Stenotrophomonas* sp. These closest-reference assignments provided a taxonomic layer for interpreting the core-genome phylogeny and subsequent capsule-associated variation.

Mapping experimentally determined capsule production onto the core-genome phylogeny revealed a partially structured phenotype distribution (Fig. 2). Capsule-positive isolates were enriched in several neighboring branches but were not limited to a single terminal clade or assigned taxon. This pattern indicated that capsule formation is not explained by species-level assignment alone and suggested that capsule-associated accessory loci may vary across related phylogenetic backgrounds.

We next screened the genomes for capsule-associated determinants using Virulence Factor Database (VFDB) and CapsuleFinder annotations. This analysis was motivated by previous work reporting capsule-associated genes in the *S. maltophilia* complex as part of a broader VFDB-based virulence-factor survey (9). Consistent with this, we detected multiple VFDB capsule-category homologs across the analyzed genomes (Table S2). However, many of these hits represented general polysaccharide-related functions, including nucleotide-sugar biosynthesis, precursor metabolism, and carrier-associated pathways, and were present in both capsule-positive and capsule-negative isolates. Therefore, individual VFDB capsule-category hits alone were insufficient to explain the capsule phenotype.

To make the VFDB/CapsuleFinder screen interpretable, related product annotations were collapsed into broader functional labels before visualization. The phylogeny-associated distribution map separated widely distributed polysaccharide-related functions from a smaller set of capsule-associated functions that were more frequent among capsule-positive isolates (Fig. 2). The broadly distributed layer included enzymes involved in UDP-sugar metabolism, phosphosugar conversion, GDP-mannose-derived sugar modification, and lipid-carrier precursor synthesis. These functions are compatible with general polysaccharide biosynthesis, but their presence in both capsule-positive and capsule-negative isolates indicates that they are not capsule-specific markers by themselves.

In contrast, capsule-positive isolates were enriched for functions more directly related to capsule biosynthesis or export, including lipid-carrier initiating glycosyltransferases, Wzc/Etk-like export-regulatory proteins, Wzb-like regulatory phosphatases, outer-membrane or periplasmic polysaccharide export components, Pap2-family phosphatases, and selected glycan-modification enzymes. Thus, the capsule phenotype was associated less with individual broad VFDB capsule-category homologs and more with the combined presence of capsule-associated biosynthesis and export functions.

### Genomes of *S. maltophilia* complex contain variable capsule-associated loci

**Fig 3:**
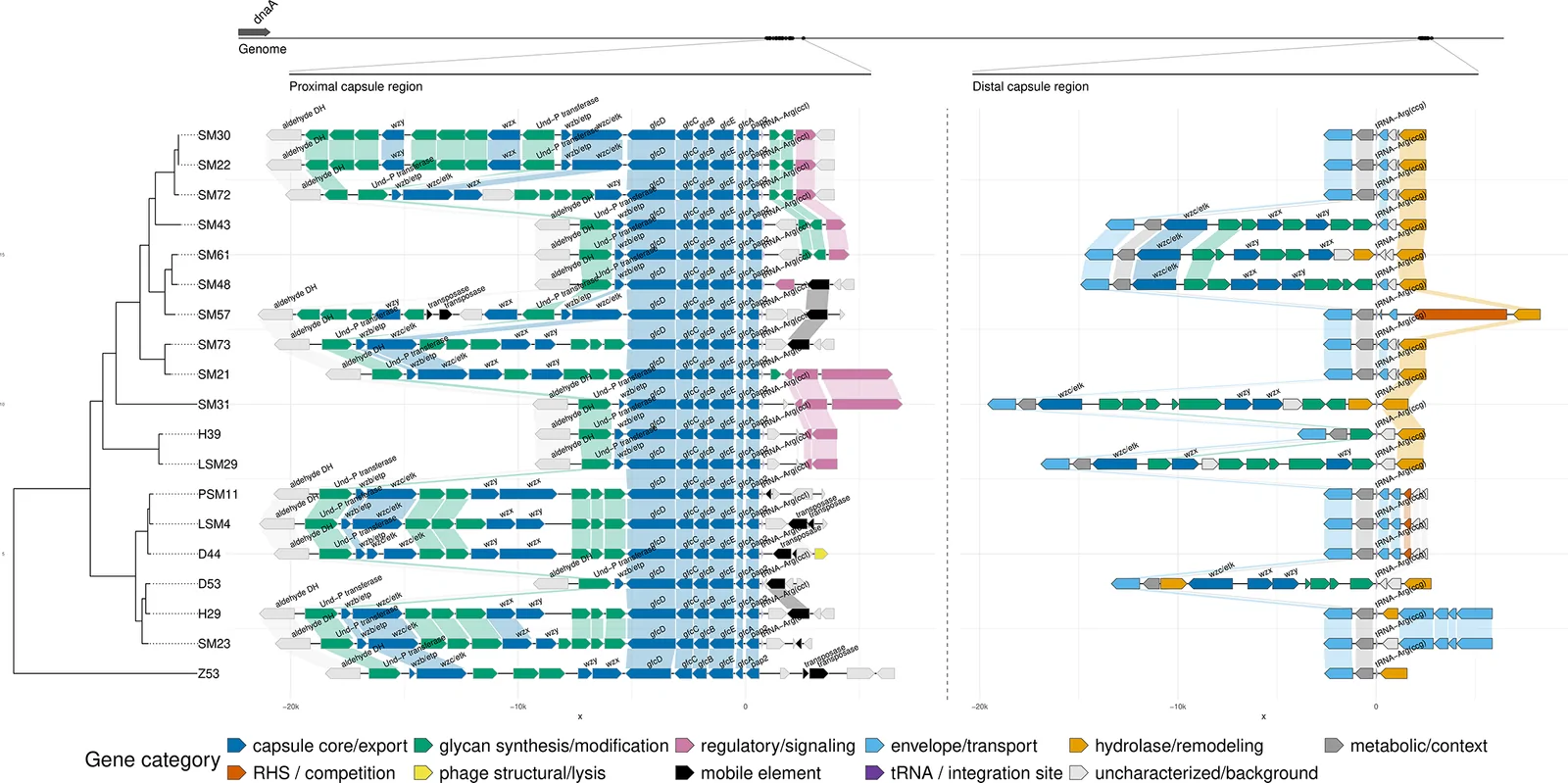
Chromosomal organization of proximal and distal capsule-associated regions in the *S. maltophilia* complex isolates. Manually curated capsule-associated regions are shown for capsule-positive isolates and the capsule-negative isolate SM57. Isolates are ordered according to the midpoint-rooted core-gene phylogeny shown on the left. The schematic above the gene maps shows the chromosomal positions of the proximal and distal regions, using *dnaA* as a reference; points indicate region midpoints across genomes. The proximal region is associated mainly with tRNA-Arg(cct), and the distal region is associated with tRNA-Arg(ccg). Genes are colored by curated functional category. Shaded polygons between adjacent rows connect homologous genes assigned to the same pangenome orthologous group. Gene labels show representative annotations used for the curated locus map.

Because gene-level annotation alone could not determine whether capsule-associated functions were genetically linked, we next examined the genomic neighborhoods of candidate capsule genes in capsule-positive and capsule-negative isolates. This allowed us to test whether VFDB and CapsuleFinder hits occurred as co-localized gene sets with features expected for capsule biosynthesis regions. The organization varied among capsule-positive isolates. In some strains, candidate genes formed a more contiguous capsule-associated region, whereas in others they were distributed between two distant tRNA-linked chromosomal regions (Fig. 3). The proximal region was associated mainly with tRNA-Arg(cct), whereas the distal region was associated with tRNA-Arg(ccg). Across these arrangements, one component was comparatively stable and contained Gfc- and Wzb/Etp-associated export/assembly functions, together with a recurrent PAP2-family phosphatase, whereas the more variable component included Wzc/Etk-, Wzx/Wzy-, and glycan-biosynthesis functions. This variable component was positioned either adjacent to the conserved proximal block or within the distal tRNA-linked region.

Capsule-negative isolates helped distinguish the capsule-associated signal from the conserved chromosomal background. Most retained the proximal and distal tRNA-linked regions together with conserved homologous flanking genes but lacked the complete architecture observed in capsule-positive isolates (Fig. S2). SM57 was the exception. It retained a proximal region closely resembling the capsule-positive organization, including Wzb/Etp-like, Wzc/Etk-like, Wzx/Wzy-like, Gfc-like, and PAP2-family genes. However, this region was interrupted by transposase insertions (Fig. 3), providing a plausible explanation for the absence of capsule production despite retention of several capsule-associated homologs.

Sequence- and structure-based clustering supported the distinction between conserved and variable components (Table S3). Gfc-like proteins, Wzb/Etp-like phosphatases, Wzc/Etk-like proteins, and several recurrent proximal proteins formed comparatively coherent groups. In contrast, Wzx-like and Wzy-like candidates were more heterogeneous. Wzx-like proteins shared polysaccharide translocation/export-related annotations and structural similarity but separated into several sequence groups, whereas Wzy-like candidates included divergent proteins annotated as oligosaccharide repeat-unit polymerases, O-antigen ligase-like proteins, EpsG-family proteins, or related polysaccharide-processing proteins. PAP2-family proteins were retained as recurrent accessory phosphatase candidates within the proximal block but were not treated as stand-alone capsule markers.

Overall, capsule production was most closely associated with the integrity of a variable multigene architecture rather than with isolated VFDB/CapsuleFinder hits or conserved flanking context alone. The most stable component was the proximal Gfc/Wzb-Etp-associated export/assembly block, while Wzc/Etk-, Wzx/Wzy-, and glycan-biosynthesis functions were more variable and could occur either proximally or within the distal tRNA-linked region. Both tRNA-associated regions were embedded within broader variable intervals containing transposases, integrases or recombinases, phage-related genes, RHS/immunity-associated genes, and other accessory functions while being surrounded by conserved homologous background genes (Fig. 3; Fig. S2). This arrangement is consistent with a recombinogenic genomic-island-like context, in line with previous reports that bacterial capsule loci can act as sites of recombination and surface-polysaccharide diversification (27).

The curated gene content was also consistent with a group IV-like, Wzx/Wzy-dependent surface-polysaccharide system, similar to O-antigen capsule systems described in *Escherichia coli* and other bacteria (28). The strongest support for this interpretation came from the recurrent Gfc-like export/assembly genes, together with Wzb/Etp- and Wzc/Etk-like regulatory components and Wzx/Wzy-like polysaccharide-processing functions. These features support the interpretation of the identified regions as capsule-associated rather than generic extracellular polysaccharide loci. Because the *S. maltophilia* complex regions were not organized as a single canonical operon, this classification should be interpreted as functional and comparative rather than as a direct one-to-one assignment to the *E. coli* group IV capsule locus.

### Representative capsule-associated loci are required for polysaccharide capsule formation

To assess whether the identified capsule-associated loci were indeed associated with the presence of the polysaccharide capsule, they were subjected to mutagenesis analyses. For this, we selected two capsule-positive clinical isolates with different capsule-locus organizations, SM21 and SM48. In the SM21 isolate, capsule-associated genes formed a more contiguous proximal region, whereas in SM48, capsule-associated genes were distributed between proximal and distal loci (Fig. 3). A double homologous crossover method using a suicide plasmid was used to create a large capsule-associated locus deletion (~6.5 kb) in the SM21 isolate and a *wzc* gene deletion (~2.2 kb) in the SM48 isolate (Fig. S3). The generated mutant isolates were named SM21ΔCPS and SM48Δ*wzc*, respectively. Whole-genome sequencing confirmed the intended deletions without detectable additional genome perturbations. The absence of a polysaccharide capsule phenotype was confirmed in both mutants by a density-dependent gradient test and SDS-PAGE fractionation with Alcian Blue staining (Fig. S4B and C). In addition to the loss of the high-molecular-weight capsular material, deletion of *wzc* in isolate SM48 resulted in a reduction of lower-molecular-weight sugars (Fig. S4). Of note, polysaccharide capsule-deficient isolates showed no substantial growth defects in Luria-Bertani broth (LB) compared to their wild-type counterparts (Fig. S4D).

**Fig 4:**
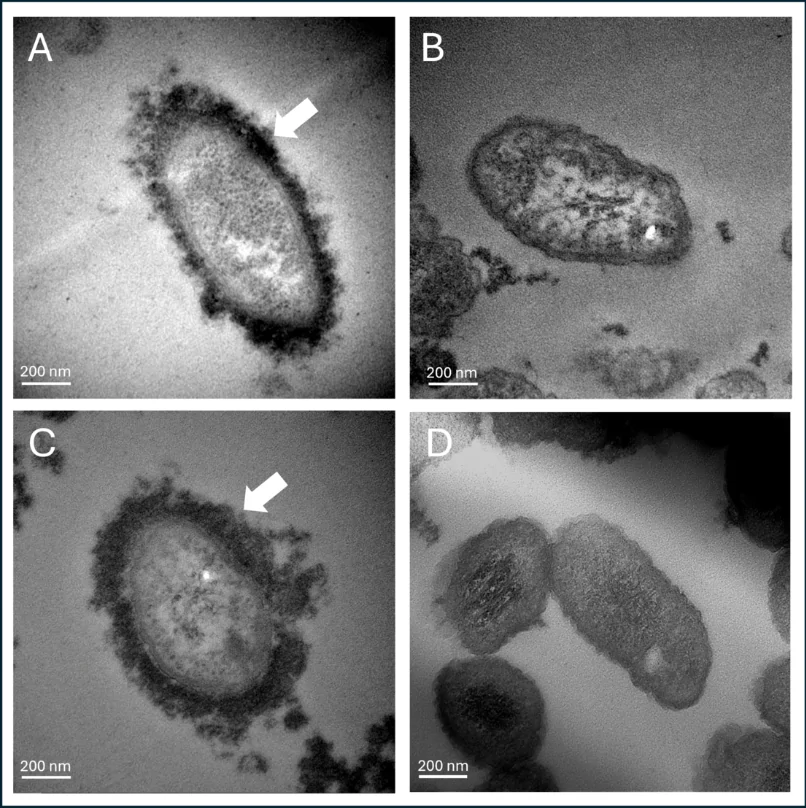
Visualization of the polysaccharide capsule of *S. maltophilia* complex isolates using TEM. Representative TEM images of (**A**) the capsulated isolate SM21, (**B**) the polysaccharide capsule-deficient mutant SM21ΔCPS, (**C**) the capsulated isolate SM48, and (**D**) the polysaccharide capsule-deficient mutant SM48Δ*wzc*. White arrows indicate the polysaccharide capsule.

To confirm that isolates of the *S. maltophilia* complex display a bona fide polysaccharide capsule, as opposed to a coat of polysaccharides not attached to the bacterial surface, we have performed transmission electron microscopy (TEM) on ultrathin sections of the bacteria. TEM analyses confirmed that isolates of the *S. maltophilia* complex display a polysaccharide capsule (Fig. 4A and C), and polysaccharide capsule synthesis was associated with the targeted capsule-associated loci, since the respective capsule-deficient mutants did not display a similar surface structure (Fig. 4B and D).

Additionally, we randomly selected seven capsulated and two non-capsulated isolates (as determined from our previous density-dependent gradient test) and visualized their polysaccharide capsule by TEM using an identical sample preparation method. All analyzed capsulated *S. maltophilia* complex isolates displayed a similar surface structure of the polysaccharide capsule (Fig. S5A), while in the non-capsulated isolates, the surface structure was not detected (Fig. S5B). Overall, these observations demonstrate that the targeted capsule-associated loci are required for polysaccharide capsule formation in the representative SM21 and SM48 backgrounds. Together with the broader TEM analysis, these results confirm the presence of a bona fide polysaccharide capsule in isolates previously identified as capsule positive by density-gradient and SDS-PAGE analyses.

### Polysaccharide capsule of *S. maltophilia* complex protects from the components of host innate immunity

**Fig 5:**
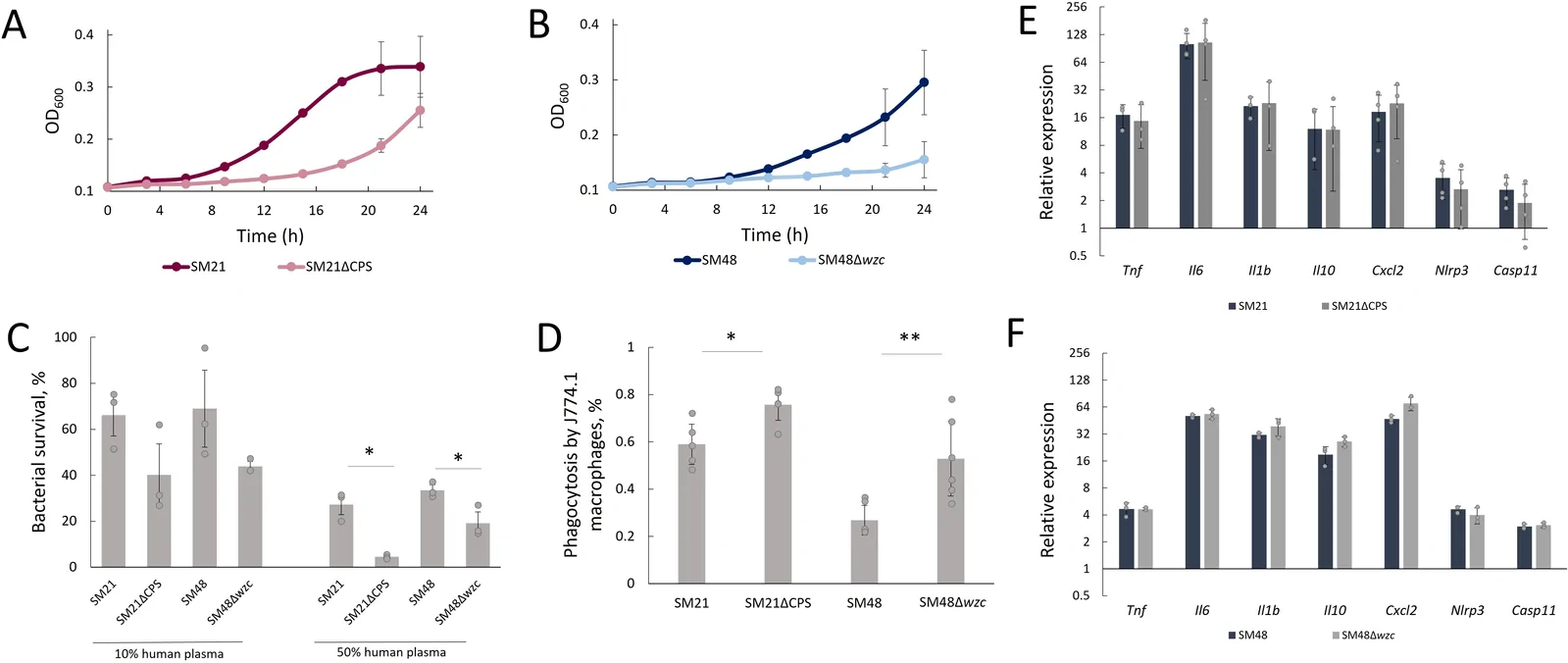
Role of polysaccharide capsule of *S. maltophilia* complex in protection from host innate immunity components and induction of inflammation. (**A**) Analysis of capsulated isolate SM21 and its capsule-deficient mutant SM21ΔCPS growth in 10% human plasma. (**B**) Analysis of capsulated isolate SM48 and its capsule-deficient mutant SM48Δ*wzc* growth in 10% human plasma. (**C**) Analysis of capsulated isolates (SM21 and SM48) and their capsule-deficient mutant (SM21ΔCPS and SM48Δ*wzc*) survival in 10% and 50% of human plasma. (**D**) Analysis of capsulated isolates (SM21 and SM48) and their capsule-deficient mutant (SM21ΔCPS and SM48Δ*wzc*) phagocytosis by J774.1 macrophages. (**E**) The relative expression of inflammation-related genes in J774.1 macrophages, induced by the capsulated isolate SM21 and its capsule-deficient mutant SM21ΔCPS. (**F**) The relative expression of inflammation-related genes in J774.1 macrophages, induced by the capsulated isolate SM48 and its capsule-deficient mutant SM48Δ*wzc*. Relative change in gene expression was calculated using the ΔΔCT method, with the actin gene expression serving as a reference. Results are expressed as the average of data from at least three biological experiments; error bars show standard deviation. *P < 0.05, **P < 0.01, as determined by the Welch *t*-test or the Mann–Whitney *U* test.

After establishing that *S. maltophilia* complex bacteria can produce a polysaccharide capsule and identifying the capsule-associated loci involved in its synthesis, we further analyzed its biological role. Polysaccharide capsule is recognized for its role in protecting other bacterial pathogens from the host immune system, especially the complement system (29) and phagocytosis (30). To assess the role of the polysaccharide capsule in the defense against blood plasma proteins, we compared the growth and survival of capsulated *S. maltophilia* complex isolates and their capsule-deficient mutants in a medium supplemented with 10% human blood plasma (Fig. 5A and B). While all strains exhibited similar growth patterns in a standard LB (Fig. S4D), capsulated isolates showed significantly enhanced growth in a plasma-containing medium compared to capsule-deficient mutants (Fig. 5A and B). Survival assays using 10% and 50% human plasma confirmed that capsulated isolates were more resistant to blood plasma-mediated killing (Fig. 5C), thus showing that the polysaccharide capsule of *S. maltophilia* complex protects from blood plasma-mediated bacterial inhibition.

Results of phagocytosis by the J774.1 murine macrophage cell line showed that capsulated isolates SM21 and SM48 were significantly more resistant to macrophage phagocytosis compared to their non-capsulated mutants (Fig. 5D), confirming that the polysaccharide capsule of the *S. maltophilia* complex also protects from phagocytosis by macrophages.

The polysaccharide capsule of other bacterial pathogens can mask bacterial surface antigens, modulating host immune responses and influencing cytokine release, thereby disrupting the coordination of host cell-mediated immunity (10). To determine whether the polysaccharide capsule of *S. maltophilia* complex influences the innate immune response, we assessed induced inflammation in J774.1 macrophages by capsulated and capsule-deficient isolates. To this end, we investigated the expression of pro-inflammatory cytokines, pyroptosis-associated caspase Casp11, and the inflammasome component NLRP3 during infection (Fig. 5E and F). Capsulated *S. maltophilia* complex isolates and their capsule-deficient mutants induced gene expression of the pro-inflammatory cytokines TNF-α, IL-6, IL-1β, the anti-inflammatory cytokine IL-10, the chemokine MIP-2, the inflammasome-associated protein NLRP3, and the non-canonical inflammasome-associated caspase-11 (Fig. 5E and F). However, the analyzed isolates did not induce gene expression of the pro-inflammatory cytokine IL-18 and the inflammasome-associated caspase-1 (data not shown). While expression of most genes was equivalent for the capsulated isolate, SM48, and the capsule-deficient mutant, SM48Δ*wzc*, the mutant showed a tendency to induce a higher expression of the MIP-2 and IL-10 coding genes, *Cxcl2* and *Il10* (Fig. 5F).

### Polysaccharide capsule protects *S. maltophilia* complex bacteria from desiccation but inhibits biofilm formation and adhesion to epithelial cells

A polysaccharide capsule can enhance bacterial adhesion to biotic and abiotic surfaces by increasing the particle contact area and providing an adherent polymer (31). However, it can also mask adhesion proteins on the bacterial surface, thus inhibiting attachment to surfaces (19), suggesting that the capsule can be used in different ways by different pathogens. Since surface adhesion is an initial step in biofilm development, the polysaccharide capsule can also play a key role in modulating the biofilm formation capacity of bacteria.

**Fig 6:**
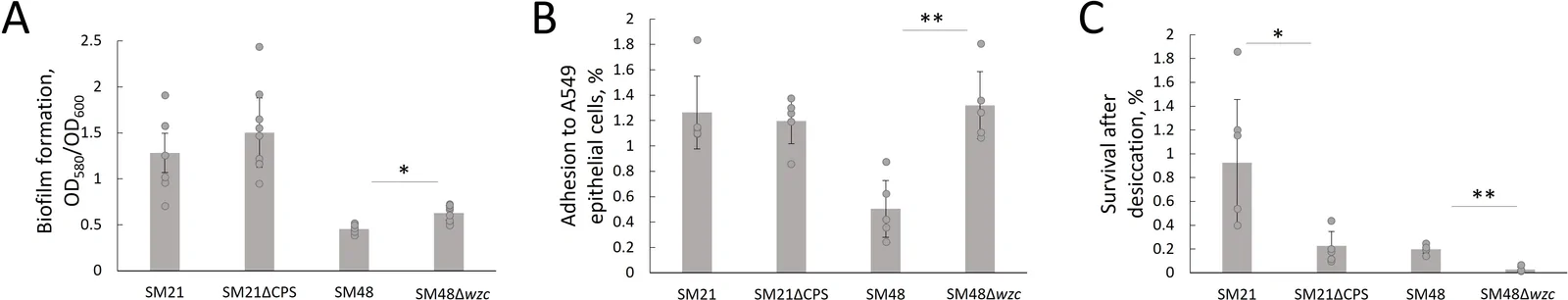
Role of *S. maltophilia* complex bacteria polysaccharide capsule in biofilm formation, adhesion to epithelial cells, and resistance to desiccation. Analysis of capsulated *S. maltophilia* complex isolates (SM21 and SM48) and their polysaccharide capsule-deficient mutants (SM21ΔCPS and SM48Δ*wzc*) in (**A**) biofilm formation on a plastic surface, (**B**) adhesion to A549 epithelial cells, and (**C**) resistance to desiccation. Results are expressed as the average of data from at least three biological experiments; error bars show standard deviation. *P < 0.05, **P < 0.01, as determined by the Welch *t*-test.

*S. maltophilia* is primarily associated with respiratory tract infections and is known to colonize the surface of human lung epithelial cells (32). To investigate the role of *S. maltophilia* complex polysaccharide capsule during the interaction with the host tissue, we assessed its contribution to adhesion to lung epithelial cells (Fig. 6B). Given that *S. maltophilia* is also notorious for forming biofilms on a variety of medical devices, especially catheters and other indwelling devices (33), we additionally examined the involvement of the polysaccharide capsule in biofilm formation on plastic surfaces (Fig. 6A).

Strikingly, the SM48Δ*wzc* mutant manifested a significant increase in both biofilm formation and adhesion to epithelial cells as compared to its wild-type strain (Fig. 6A and B). However, no effect on adhesion and biofilm formation was identified by analyzing another pair of capsulated and capsule-deficient isolates, SM21 and SM21ΔCPS. Overall, this suggests that the capsule can play an important role in *S. maltophilia* complex adhesion to both biotic and abiotic surfaces; however, since different *Stenotrophomonas* isolates displayed variation in this trait, the polysaccharide capsule may not be an exclusive factor responsible for these phenotypes.

In addition to its role in infection and adhesion, the polysaccharide capsule of other bacterial pathogens is recognized for its role in protecting from various environmental pressures by creating a barrier around the bacterial cell wall. Desiccation resistance is especially important for the survival and persistence of bacteria in a hospital environment and is known to be enhanced by forming a capsule (34–36). Analysis of capsulated and capsule-deficient *S. maltophilia* complex isolates revealed that both wild-type isolates SM21 and SM48 survived desiccation significantly better compared to their non-capsulated mutants (Fig. 6C), showing that the polysaccharide capsule protects bacteria of the *S. maltophilia* complex from desiccation.

## DISCUSSION

Bacteria belonging to the *Stenotrophomonas maltophilia* complex were recently regrouped from a single species, *S. maltophilia* (9). It represents an emerging group of opportunistic pathogens of increasing clinical significance; however, the knowledge of their virulence mechanisms remains limited. The intrinsic multidrug resistance of the *S. maltophilia* complex significantly restricts treatment options, with trimethoprim-sulfamethoxazole currently considered the drug of choice (37). However, rising antibiotic resistance rates, as well as the biofilm formation ability typical to *Stenotrophomonas*, indicate the need for alternative treatment strategies and require a deeper understanding of their mechanisms of pathogenesis.

In this study, we have investigated a previously uncharacterized virulence-associated trait of *S. maltophilia* complex bacteria: the polysaccharide capsule. An extensive bioinformatic study of capsule synthesis genes across diverse bacterial species has been previously conducted, but it included only a small number of *S. maltophilia* genomes (11), in which specific genes required for polysaccharide capsule synthesis were not found. However, our previous work indicated that part of the *S. maltophilia* isolates form a putative polysaccharide capsule (20), and a more recent work by Li et al. identified several capsule-related genes encoded in the genomes of *S. maltophilia* complex isolates (9). In this study, we analyzed a large collection of clinical (*n* = 105) and environmental (*n* = 42) isolates from Lithuania and showed that bacteria from the *S. maltophilia* complex possess a bona fide polysaccharide capsule. We confirmed the presence of a polysaccharide capsule in *S. maltophilia* complex bacteria by several independent methods: polysaccharide fractionation by SDS-PAGE and staining with Alcian Blue, polysaccharide capsule identification by the density-dependent gradient, and capsule visualization using TEM. While all these methods could consistently distinguish between capsulated and non-capsulated isolates of the *S. maltophilia* complex, the density-dependent gradient test would be the most time- and resource-efficient and yet reliable method for quick large-scale screening in the future.

Furthermore, we identified capsule-associated genomic regions in capsule-producing *S. maltophilia* complex isolates. Capsule-positive isolates carried recurrent capsule-associated gene sets, although their organization varied among strains. In some isolates, these genes formed a more contiguous proximal region, whereas in others they were split in two and distributed between proximal and distal chromosomal regions. These loci were detected across multiple assigned lineages of the *S. maltophilia* complex, rather than being restricted to a single species-level group. One capsule-negative isolate retained a capsule-like proximal region, but this region contained transposase insertions, providing a plausible explanation for the absence of capsule production. The curated gene content was most consistent with a group IV-like, Wzx/Wzy-dependent surface-polysaccharide system and included homologs of the Gfc-associated capsule export/assembly machinery previously described in O-antigen capsule systems of *E. coli*, *Aeromonas hydrophila*, and *Shigella sonnei* (38). However, the organization of the *S. maltophilia* complex loci differed from canonical *E. coli* group IV capsule loci. Further structural and biochemical analyses of capsule composition and linkage will be required to determine whether these isolates produce an O-antigen-type capsule. Such studies will also be necessary to characterize the additional surface-associated carbohydrates and potential capsule biosynthesis intermediates affected by *wzc* deletion in strain SM48, detected by Alcian Blue staining.

The requirement of representative capsule-associated loci for capsule formation was supported by deletion analysis: SM21ΔCPS and SM48Δ*wzc* lost the capsule phenotype by density-gradient analysis, Alcian Blue staining, and TEM. However, attempts to restore capsule production by replicative plasmid-based complementation were unsuccessful. The deleted region in the SM21 mutant and the *wzc* gene in the SM48Δ*wzc* mutant were first introduced within a pBAD vector and induced with arabinose. In addition, *wzc* was cloned with its putative native promoter and with the promoter of the highly expressed *rpoB* gene derived from the corresponding *S. maltophilia* complex isolate. None of these approaches resulted in recovery of the capsule-positive phenotype (data not shown). This may reflect the complexity of the capsule-associated loci identified in this study, as capsule production appears to be associated with a multigene architecture containing biosynthetic, export/assembly, and regulatory components, rather than with a single isolated determinant. Restoration of capsule formation may therefore require native chromosomal context, appropriate gene dosage, and balanced expression of multiple envelope-associated components. This interpretation is consistent with studies of other capsule and surface-polysaccharide systems, in which capsule formation depends on the coordinated activity of glycan-biosynthesis enzymes, polymerization/export proteins, and Wzc/Wzb-like or CpsD/CpsB-like regulatory components (28, 39–41). Moreover, incomplete or failed plasmid-based complementation has been reported for capsule-associated mutants, including a *Streptococcus iniae cpsD* mutant, where constitutive plasmid expression did not restore the wild-type capsule-associated phenotype, and inducible expression restored the phenotype only over a narrow induction range (42). Therefore, we believe that restoration of the gene at its native chromosomal locus may represent a more suitable approach for future complementation studies than plasmid-based expression.

The organization of the capsule-associated regions suggests that capsule production in the *S. maltophilia* complex is encoded by a modular and evolutionarily dynamic locus architecture rather than by a single conserved operon. The most conserved component was the proximal Gfc/Wzb-Etp-associated block, consistent with a role in capsule export or surface assembly. In *E. coli*, group IV/O-antigen capsule formation requires the *gfcABCDE-etp-etk* locus, which encodes proteins implicated in capsule export and surface expression (39). Etk and Etp correspond to Wzc-like tyrosine kinase and Wzb-like phosphatase components, respectively, and cycling of their phosphorylation states is required for group IV capsule formation (40). More recent biochemical and cell-envelope localization work supports a role for Gfc proteins, particularly GfcB, GfcC, and GfcD, in the outer-membrane/periplasmic assembly of the group IV capsule (28). In contrast, glycan repeat-unit identity in Wzx/Wzy-dependent surface-polysaccharide systems is determined by more variable initiating transferases, glycosyltransferases, Wzx/Wzy-like proteins, and sugar-modification enzymes (43). Thus, the organization observed here is consistent with a comparatively conserved export/assembly module combined with a more variable biosynthetic and modification layer.

The recurrent association of the capsule-linked regions with homologous flanking genes, broadly similar chromosomal positions relative to *dnaA* and specific tRNA genes, indicates that these loci occupy conserved chromosomal neighborhoods that have undergone local remodeling. tRNA genes are frequent integration sites for prokaryotic genetic elements (44), and genomic islands commonly occur adjacent to tRNA genes and carry genes acquired by horizontal transfer (45). Comparable remodeling of surface-polysaccharide loci has been described in *Shigella flexneri*, where serotype-converting bacteriophages and plasmids encode O-antigen glycosylation, acetylation, and phosphoethanolamine-modification functions that generate antigenic variation (46). The mobile-element-rich context of the *S. maltophilia* complex loci, together with the structural similarity of several glycan-biosynthesis and sugar-modification proteins to viral or bacteriophage proteins, is therefore consistent with exchange between capsule-associated loci and mobile-element-associated gene pools. These observations support a role for horizontal exchange in shaping the variable glycan-biosynthesis and modification layer, but they do not resolve the evolutionary origin of individual locus components. Addressing this question would require dedicated gene-by-gene phylogenetic analyses and broader comparative sampling, which are beyond the scope of the present study. Because bacterial surface polysaccharides can act as phage receptors or as barriers to receptor access, phage-mediated selection may also have contributed to the diversification of the variable glycan-modification and polymerization components. This model is consistent with broader evidence that bacterial capsule and surface-polysaccharide loci can act as hotspots of recombination and antigenic diversification (27).

In bacterial pathogens, the presence of a polysaccharide capsule is known as a non-universal (accessory) virulence trait. While capsular structures are well recognized for their roles in immune evasion (10), their occurrence is not universal and is usually associated with specific bacterial species or isolates. Although the polysaccharide capsule is mostly analyzed as a trait of bacterial pathogens, in this study, we have shown that in the *S. maltophilia* complex, the isolates of environmental origin were twice as likely to possess one as compared to the isolates of clinical origin. A higher prevalence of capsule-associated genes has already been observed in environmental and commensal bacteria as compared to pathogenic ones (11). The polysaccharide capsule was thus proposed to protect from a variety of physical and chemical stresses (e.g., desiccation, antibiotics, and bacteriocins), increasing the environmental range of bacteria and making them more resilient to perturbations (11, 47). A related recent observation comes from a single-isolate study of the drinking-water *S. maltophilia* complex genome, which identified a candidate EPS/envelope-remodeling genomic island containing *wzc*, *ugd*, *algL*, and a GT26-family glycosyltransferase. However, capsule production was not experimentally tested, and the functional role of this island remains to be determined (48). In this study, we have shown that the polysaccharide capsule of *S. maltophilia* complex bacteria protects them from desiccation, consistent with the findings of the polysaccharide capsule function in other bacterial pathogens (36, 49, 50). However, we also determined that possessing a capsule has a negative effect on the formation of biofilm and adhesion to surfaces, likely due to the masking of bacterial surface proteins responsible for attachment. It is known that flagella (51) and fimbriae (52) play an important role in the adhesion of *S. maltophilia* to surfaces and subsequent biofilm formation. Partial coverage of these structures by a polysaccharide capsule could influence their functionality, as was shown in the case of *Klebsiella pneumoniae* (53). Also, in *Escherichia coli*, it has been demonstrated that the polysaccharide capsule can inhibit the function of short adhesins (19), and this phenomenon is likely to extend to other gram-negative bacteria. Unfortunately, very little is known about the shorter surface proteins of *S. maltophilia* complex bacteria. Thus, it is unclear whether they could influence the differences in adhesion and biofilm formation observed between wild-type and capsule-deficient *S. maltophilia* complex.

To assess the particular importance of the polysaccharide capsule in the *S. maltophilia* complex, we compared the survival abilities of capsulated and capsule-deficient clinical isolates in relation to infection-related host defense mechanisms. A polysaccharide capsule can mask the bacterial surface and its surface-associated proteins, thereby protecting it from detection by phagocytic cells (13). By masking epitopes of antibodies on the bacterial surface, the capsule can also block the activation of the complement system or prevent the proper insertion of the membrane attack complex, thereby inhibiting bacterial lysis (54). Bacterial capsules are also typically hydrophilic and carry a negative charge. This characteristic reduces susceptibility to phagocytosis, as negatively charged polysaccharides on the bacterial surface repel the similarly charged surface of phagocytes, creating an unfavorable interaction that hinders efficient phagocytosis (15). In this work, we have shown that the polysaccharide capsule protects *S. maltophilia* complex bacteria from both phagocytosis and plasma-mediated killing, emphasizing its importance in the defense from the major components of host innate immunity. The polysaccharide capsules are also known to act as immunomodulatory molecules in the host–bacteria interactions (55). Polysaccharides can influence the immune system by engaging with pattern recognition receptors or altering immune cell signaling (56, 57). However, the ability of *S. maltophilia* complex bacteria to trigger an immune response remains poorly understood. Since opportunistic bacteria infect immunosuppressed patients, the pro-inflammatory response of innate immunity components is particularly important for effectively controlling the infection (58). Previous studies have shown that the outer membrane vesicles released by *S. maltophilia* induced the expression of pro-inflammatory cytokine and chemokine genes, such as IL-1β, IL-6, IL-8, TNF-α, and MCP-1, in A549 cells, as well as in a mouse infection model *in vivo* (59). Our data revealed that macrophages also induced the production of pro-inflammatory cytokines and chemokines, such as TNF-α, IL-6, and MIP-2, as well as the pyroptosis-associated cytokine IL-1β. These findings are consistent with previous research demonstrating the induction of pro-inflammatory cytokines using mouse infection models *in vivo* (60, 61). The results of our study also showed that the absence of a polysaccharide capsule led to a tendency toward higher expression of the MIP-2 chemokine gene, a key mediator of neutrophil recruitment (62), and IL-10, the anti-inflammatory cytokine (63). Our observation is in agreement with other studies that have shown similar inhibitory effects of polysaccharide capsules on MIP-2 and IL-10 expression in other bacterial pathogens (64, 65). However, as a tendency in the difference of MIP-2 and IL-10 expression was only observed by analyzing one pair of wild-type and capsule-deficient strains (similar to biofilm formation and adhesion), it suggests that modulation of the host immune response and suppression of inflammation might be isolate dependent.

To conclude, in this study, we identified and characterized the polysaccharide capsule as a novel virulence-associated trait of *S. maltophilia* complex bacteria. We demonstrated that the polysaccharide capsule protects *S. maltophilia* complex bacteria from the host immune system’s defense mechanisms; however, in certain isolates, it also confers a disadvantage in biofilm formation and adhesion.

## MATERIALS AND METHODS

### Bacteria used in this study

Isolates used in this study are listed in Table S4. In total, 105 clinical *S. maltophilia* complex isolates were retrieved from biological samples of patients from several hospitals in Lithuania, and 42 environmental *S. maltophilia* complex isolates were collected from the environment (soil and fish gut). All isolates had been provisionally specified as *S. maltophilia* by conventional laboratory techniques. Clinical strains were assigned to the species using matrix-assisted laser desorption ionization-time-of-flight mass spectrometry method (MALDI BioTyper, Bruker). In addition, the environmental strains were first screened using the *Stenotrophomonas*-specific primers SM1/SM4 and subsequently confirmed by 16S rRNA gene sequencing using ≥99% sequence identity as the species threshold (66).

For plasmid construction and cloning, the *Escherichia coli* JM107 strain (67) was used.

### Bacterial genome mutagenesis

Bacterial genome mutagenesis was performed using a double homologous crossover method using a suicide plasmid, as described before (68). Briefly, around 1 kb upstream and downstream regions of targeted genes, together with the chloramphenicol resistance gene, were amplified using Phusion Plus PCR Master Mix (Thermo Fisher Scientific), and amplified fragments were cloned into the suicide plasmid pET19_*sacB* (68) using NEBuilder HiFi DNA Assembly kit (New England Biolabs). Plasmids were transferred to *S. maltophilia* complex isolates using electroporation, as described before (69). Colonies with integrated plasmids were selected on Luria-Bertani broth agar (LBA) with 30 mg/L chloramphenicol and additionally identified by PCR. Plasmid integration-positive colonies were grown on LBA containing 10% sucrose, mutants were selected by PCR, and mutations were verified by Sanger sequencing. To check whether the created capsule-locus mutants had acquired additional genome mutations, full genome sequencing using Oxford Nanopore technology was performed.

### Analysis of polysaccharide capsule production using the density-dependent gradient test

Gradient test analysis was performed as previously reported (70), with some modifications. Briefly, bacterial cultures were grown in LB overnight at 28°C (for environmental isolates) or 37°C (for clinical isolates) for 20 h (with shaking). Eight hundred microliters of bacterial overnight culture was centrifuged at 5,000 × *g* for 2 min; the bacterial pellet was resuspended in 50 µL of phosphate-buffered saline (PBS). Suspension was placed on 900 µL of 50% silica colloid Percoll density-gradient medium (Cytiva Life Sciences) and centrifuged in a 1.5 mL Eppendorf tube at 3,000 × *g* for 30 min. The position of the bacterial pellet was evaluated and visualized immediately after centrifugation.

### Analysis of polysaccharide capsule production using Alcian Blue staining

Capsular polysaccharide analysis using Alcian Blue staining was performed as described earlier (20). Briefly, isolates were grown on LBA overnight at 28°C (for environmental isolates) or 37°C (for clinical isolates). Bacterial cultures were suspended in 500 µL PBS buffer at OD_600_ = 5. The polysaccharide capsule was detached from bacteria by vigorously mixing samples with a tabletop vortex mixer at maximum speed for 30 s. Samples were centrifuged at 9,000 × *g* for 10 min, detached polysaccharides in the supernatant were precipitated in 2.5 vol of ice-cold ≥99.6% ethanol and 1/10 vol of 3 M sodium acetate (pH 5.2) overnight at −20°C. Samples were centrifuged at 20,000 × *g* for 30 min (4°C), and the pellet was washed with 70% ice-cold ethanol and centrifuged at 20,000 × *g* for 30 min (4°C). The pellet was resuspended in the SDS-PAGE sample loading buffer and boiled for 5 min. Samples were fractioned in the gradient (4%–15%) SDS-PAGE gels. After electrophoresis, gels were stained overnight with 0.1% (wt/vol) Alcian Blue 8GX (Thermo Fisher Scientific).

### Visualization of the polysaccharide capsule

The polysaccharide capsule visualization was performed using TEM on ultrathin sections. Samples were prepared using Alcian Blue 8GX cationic dye (Thermo Fisher Scientific) and L-lysine acetate as previously reported (71). To preserve polysaccharide capsule structure, fixation, wash, and dehydration procedures were performed in a 12-well plate containing bacterial colonies on agar cubes, which were submerged in all buffers needed for sample preparation (71).

Ultrathin sections were obtained using a diamond knife-based Ultramicrotome EM UC7 (from Leica Microsystems); visualization was performed using a transmission electron microscope FEI Tecnai 10 (100 kV) with Veleta camera and SIS iTEM v5.1 software (Olympus).

### Biofilm formation

The biofilm-forming ability was evaluated using a crystal violet dye assay (20). Overnight cultures of bacterial isolates were grown in LB at 37°C for 20 h (with shaking). Overnight cultures were diluted 50-fold (in a total volume of 200 μL) and incubated in a U-shaped 96-well polystyrene microplate (Nerbe Plus) at 28°C or 37°C for 24 h. The OD_600_ of the planktonic culture was measured; the wells were washed with PBS (three times); and the biofilm formed was stained with 0.1% (wt/vol) crystal violet (Carl Roth) for 15 min. Residual dye was removed by washing with PBS (five times), and the biofilm-bound crystal violet was dissolved in 200 μL of 99.6% ethanol per well, followed by OD_580_ measurement. The biofilm formation capacity was expressed as OD_580_/OD_600_.

### Desiccation assay

The desiccation assay was performed as previously reported, with some modifications (35). Overnight cultures of bacterial isolates were grown in LB at 37°C for 20 h (with shaking). The optical density of overnight cultures was adjusted; bacterial cultures were suspended in PBS at OD_600_ = 7.5; and samples were plated on LBA to determine the number of CFU. Ten microliters of bacterial suspension was placed in a 96-well F-shaped microplate; drops were dried in a laminar flow cabinet at room temperature and further incubated at 28°C for 24 h. After incubation, samples were resuspended in PBS and plated on LBA to determine the number of CFU. The percentage of surviving bacteria was determined by comparing the CFU of samples before and after desiccation.

### Growth and survival in human plasma

Human peripheral blood from healthy donors was collected into a vacutainer with ethylenediaminetetraacetic acid (Greiner Bio-One). Human plasma was extracted from blood by centrifugation at 1,800 rpm for 20 min. Plasma was additionally centrifuged at 3,800 rpm for 20 min to remove platelets.

To assess blood serum-mediated growth inhibition, overnight cultures of *S. maltophilia* complex isolates were grown in LB at 37°C for 20 h (with shaking) and diluted 100-fold into LB supplemented with 10% human plasma (total volume of 200 μL). Bacterial isolates were grown in a 96-well F-shaped plate, and the growth was measured by the Infinite M200 Pro plate reader as OD_600_ at 37°C for 24 h (with shaking).

To evaluate blood serum-mediated bacterial killing, overnight cultures of bacterial isolates were grown in LB at 37°C for 20 h (with shaking); 5 × 10^6^ CFU of isolates were incubated in 10% and 50% of blood plasma for 1 h at 37°C; and samples before and after incubation were plated on LBA to determine CFU. The efficiency of blood serum-mediated killing was determined by comparing the CFU of bacteria before and after incubation with blood plasma.

### Resistance to phagocytosis

Bacterial resistance to phagocytosis was evaluated using the J774.1 macrophage cell line (72), as previously reported (35). Macrophages were grown in Dulbecco’s modified Eagle medium (DMEM + GlutaMAX) (Gibco) supplemented with 10% heat-inactivated fetal bovine serum (FBS) (Gibco) at 37°C with 5% CO_2_. J774.1 cells were plated in a 96-well plate at a density of 10^5^ cells per well and incubated overnight. Overnight cultures of bacterial isolates were grown in LB at 37°C for 20 h (with shaking) and washed with PBS (two times), and 2 × 10^6^ bacteria were used for infections in each well (the number of each isolate CFU was verified by plating samples on LBA). Infected macrophages were incubated at 37°C for 1 h. After infection, macrophages were washed with PBS (three times). To kill non-phagocytosed bacteria, macrophages were incubated with 400 μg/mL gentamicin for 30 min and later washed with PBS (three times). To determine phagocytic activity, macrophages were lysed using double-distilled H_2_O with intense pipetting; bacteria were plated on LBA to determine the CFU of bacteria inside macrophages. The efficiency of macrophage phagocytosis was determined by comparing the CFU of initial bacterial samples and the CFU after lysis of the macrophages.

### Adhesion to lung epithelial cells

Adhesion to host cells was evaluated using the A549 human epithelial cell line (73), as described before (35). For adhesion experiments, A549 cells were grown in DMEM medium supplemented with 10% heat-inactivated FBS at 37°C with 5% CO_2_. A549 cells were plated in a 96-well plate at a density of 10^5^ cells per well and incubated overnight. Overnight cultures of bacterial isolates were grown in LB at 37°C for 20 h (with shaking) and washed with PBS (two times), and 10^7^ of bacteria were used for infections in each well (the number of each isolate CFU was verified by plating samples on LBA). A549 epithelial cells were incubated with bacteria for 90 min. After incubation, non-adherent bacteria were washed with PBS (three times); A549 cells were lysed using double-distilled H_2_O with intense pipetting; and bacteria were plated on LBA to determine CFU. The efficiency of bacterial adhesion to epithelial cells was determined by comparing the CFU of initial bacterial samples and the CFU after incubation.

### Analysis of host inflammatory response

J774.1 macrophage cells were grown in DMEM with 10% heat-inactivated FBS at 37°C with 5% CO_2_. J774.1 cells were plated into 12-well plates at a density of 8 × 10^5^ cells per well and incubated overnight. Overnight cultures of bacterial isolates were grown in LB at 37°C for 20 h (with shaking) and washed with PBS (two times), and 8 × 10^7^ of bacteria per well were used for the infections (the number of each isolate CFU was verified by plating samples on LBA). A549 and J774.1 cells were incubated with bacteria for 2 h at 37°C. Cells were washed with PBS (two times), detached from plastic using a scraper, centrifuged at 500 × *g* for 10 min at room temperature, and kept at −80°C until RNA isolation.

RNA was isolated using the GeneJET RNA Purification Kit (Thermo Fisher Scientific), and cDNA was synthesized using Maxima H Minus First Strand cDNA Synthesis Kit with dsDNase (Thermo Fisher Scientific), using the oligo(dT)_18_ primer for cDNA synthesis. Gene expression was evaluated by qPCR using DreamTaq polymerase with SYTO-9 fluorescent dye (both from Thermo Fisher Scientific) using CFX Real-Time System (from Bio-Rad) or Maxima SYBR Green qPCR Master Mix (Thermo Fisher Scientific) using the QuantStudio3 Real-time PCR System (from Applied Biosystems). Primers used for qPCR are listed in Table S5.

The change in gene expression was assessed using the ∆∆Ct method (74), with the actin gene serving as a reference.

### Whole-genome sequencing, assembly, annotation, and comparative genomics

Genomic DNA was extracted using the GeneJET Genomic DNA Purification Kit (Thermo Fisher Scientific) according to the manufacturer’s recommendations. DNA concentration was measured with a Qubit Flex fluorometer using the Qubit dsDNA High Sensitivity Assay Kit (Thermo Fisher Scientific), and purity was assessed spectrophotometrically with a NanoDrop One spectrophotometer (Thermo Fisher Scientific). Libraries were prepared with the Oxford Nanopore Rapid Barcoding Kit 24 v14 (SQK-RBK114.24) according to the manufacturer’s protocol. Sequencing was performed on a MinION Mk1B or a PromethION 2 Solo device using FLO-MIN114 or FLO-PRO114M flow cells (Oxford Nanopore Technologies) for up to 72 h. Raw signal data were base-called with Dorado (https://github.com/nanoporetech/dorado) using a super-accuracy model. Reads shorter than 1,000 bp were removed, and the highest-quality 95% of reads were retained using Filtlong v0.2.1.

*De novo* genome assemblies were generated from filtered Oxford Nanopore reads using Autocycler (75), which combines multiple independent long-read assemblies into a consensus assembly. The workflow included several bacterial long-read assemblers, including Canu (76), Flye (77), metaMDBG (78), Raven (79), miniasm (80), NECAT (81), NextDenovo (82), and wtdbg2 (83). Autocycler consensus assemblies were reoriented with dnaapler v1.3.0 (84) using the all workflow and converted from GFA to FASTA format with autocycler gfa2fasta. Genome annotation was performed with Bakta v1.11.4 (85) using the full Bakta database.

Taxonomic classification of the assembled genomes was performed with GTDB-Tk v2.4.0 (86) using the GTDB r226 reference database and the classify_wf workflow. In parallel, assemblies were compared with skani v0.3.1 (87) against a custom reference database constructed from the species framework of Chauviat et al. (26). Both GTDB-Tk classifications and skani-based assignments were retained for species-level interpretation.

A pangenome was constructed from Bakta-annotated genomes using PGAP2 v1.1.0 (88). Input GFF files were analyzed with pgap2 main, and a core-gene phylogeny was inferred from the PGAP2 output using pgap2 post singletree with IQ-TREE as the tree-building method.

### Identification of the capsule synthesis-associated loci

Capsule-associated genomic regions were identified by combining capsule-related gene detection with pangenome-guided analysis of local genomic context. Predicted proteins were screened against VFDB (89) set A and CapsuleFinder (11) profiles to identify capsule- or polysaccharide-associated genes. VFDB BLASTP hits were retained using an *E*-value threshold of <1 × 10^−10^ and a query HSP coverage of ≥70%. Hits assigned to capsule-related VFDB categories, together with CapsuleFinder matches to capsule-biogenesis components, were used as initial markers for candidate regions.

Candidate regions were not defined from marker genes alone, because individual polysaccharide-associated genes may occur in capsule, exopolysaccharide, lipopolysaccharide, or other glycan-associated contexts. Instead, genomic neighborhoods surrounding the initial markers were compared across strains using pangenome orthologous groups. Regions containing multiple capsule- or polysaccharide-associated markers in the same local neighborhood were retained for comparative analysis together with their surrounding genes. Homologous regions were recognized by shared orthologous-group composition, conserved flanking genes, and local synteny, allowing recurrent loci to be separated from unrelated genomic neighborhoods containing similar glycan-associated annotations.

The recurrent pangenome-defined contexts were then used to recover homologous regions across the full strain set, including strains in which the corresponding region did not contain a direct VFDB or CapsuleFinder capsule hit. Recovered regions were manually refined using gene order, conserved flanking orthologous groups, local synteny, and nearby tRNA-associated landmarks. This analysis resolved two major recurrent genomic regions, hereafter referred to as the tRNA-Arg(cct)-associated and tRNA-Arg(ccg)-associated regions. For both regions, the final curated loci included predicted capsule-associated genes, neighboring accessory genes, and conserved flanking genes required to define homologous locus boundaries across strains.

Proteins encoded within the curated loci were annotated using an integrated sequence-, structure-, and context-based approach. Bakta product annotations were retained where informative, and VFDB/CapsuleFinder results were incorporated for proteins with direct capsule- or polysaccharide-related matches. For additional sequence-based annotation, all proteins from the curated regions were searched by BLASTP against protein sequences corresponding to AlphaFold Database v4 (90)-predicted structures from the genus *Stenotrophomonas*. For each query, the top hit was selected by ranking matches by *E*-value and bit score; coverage values were retained to support manual interpretation of full-length versus partial similarity.

For structure-based annotation, proteins encoded within the curated loci were first linked to their corresponding AlphaFold Database v4-predicted structures. The resulting curated-region structure set was searched with Foldseek (91) in two separate analyses. First, query structures were compared against AlphaFold Database v6 (92), and compressed prediction files for the Swiss-Prot subset were used to support structure-based functional annotation. Second, the same query structures were compared against the Big Fantastic Virus Database (93) to assess possible phage- or virus-related structural similarity. Foldseek matches from both searches were evaluated using probability, TM score, alignment coverage, and consistency with sequence-based annotation and local genomic context.

Final gene labels were assigned manually by integrating Bakta annotations, VFDB and CapsuleFinder results, BLASTP similarity to *Stenotrophomonas* AlphaFold DB v4 proteins, Foldseek matches against AlphaFold DB v6 Swiss-Prot, Foldseek matches against BFVD, pangenome orthology, and local synteny. Genes were also assigned to broad functional classes for visualization and interpretation, including capsule core/export, glycan synthesis or modification, tRNA/integration-site-associated genes, mobile-element genes, regulatory or signaling genes, envelope or transport functions, hydrolase/remodeling enzymes, RHS/competition-associated genes, phage-related genes, metabolic/context genes, and uncharacterized genes. Particular attention was given to gene combinations compatible with Wza/Wzc-dependent group IV capsule or related exopolysaccharide export systems. The final curated locus annotations are provided in Table S3.

### Statistical analysis

All quantitative data were obtained from at least three biological experiments. Data normality was evaluated using the Shapiro–Wilk test. For normally distributed data, statistical significance was assessed using a two-tailed Welch’s *t*-test. When normality was not met, or when the sample size was fewer than five observations, the non-parametric Mann–Whitney *U* test was applied.

## Data Availability

The raw reads and genome assemblies generated in this study are available from the European Nucleotide Archive under accession number PRJEB96325.

## SUPPLEMENTAL MATERIAL

Fig. S1 mbio.01635-26-s0001.jpg Evaluation of the polysaccharide capsule phenotype in a collection of *S. maltophilia* complex isolates.

Fig. S2 mbio.01635-26-s0002.pdf Organization of proximal and distal capsule-associated loci.

Fig. S3 mbio.01635-26-s0003.pdf Polysaccharide capsule synthesis-related genetic operons of clinical *S. maltophilia* complex isolates.

Fig. S4 mbio.01635-26-s0004.jpg Analysis of polysaccharide capsule-expressing *S. maltophilia* complex isolates.

Fig. S5 mbio.01635-26-s0005.jpg Visualization of polysaccharide capsule of clinical and environmental isolates of *S. maltophilia* complex.

Legends mbio.01635-26-s0006.docx Legends for supplemental figures.

Table S1 mbio.01635-26-s0007.xlsx Taxonomic assignment of *Stenotrophomonas* isolates used in this study.

Table S2 mbio.01635-26-s0008.xlsx Capsule-associated genes detected by VFDB and CapsuleFinder screening.

Table S3 mbio.01635-26-s0009.xlsx Curated annotation of capsule-associated loci.

Tables S4 and S5 mbio.01635-26-s0010.docx Isolates and oligonucleotides used.

ASM does not own the copyrights to Supplemental Material that may be linked to, or accessed through, an article. The authors have granted ASM a non-exclusive, world-wide license to publish the Supplemental Material files. Please contact the corresponding author directly for reuse.

## References

[1] Chang YT, Lin CY, Chen YH, Hsueh PR. 2015. Update on infections caused by *Stenotrophomonas maltophilia* with particular attention to resistance mechanisms and therapeutic options. Front Microbiol 6:893. doi:10.3389/fmicb.2015.00893 PMC4557615 26388847

[2] Rello J, Kalwaje Eshwara V, Lagunes L, Alves J, Wunderink RG, Conway-Morris A, Rojas JN, Alp E, Zhang Z. 2019. A global priority list of the TOp TEn resistant microorganisms (TOTEM) study at intensive care: a prioritization exercise based on multi-criteria decision analysis. Eur J Clin Microbiol Infect Dis 38:319–323. doi:10.1007/s10096-018-3428-y 30426331

[3] Gröschel MI, Meehan CJ, Barilar I, Diricks M, Gonzaga A, Steglich M, Conchillo-Solé O, Scherer I-C, Mamat U, Luz CF, et al.. 2020. The phylogenetic landscape and nosocomial spread of the multidrug-resistant opportunist *Stenotrophomonas maltophilia*. Nat Commun 11:2044. doi:10.1038/s41467-020-15123-0 PMC7184733 32341346

[4] Brooke JS. 2021. Advances in the microbiology of *Stenotrophomonas maltophilia*. Clin Microbiol Rev 34:e00030-19. doi:10.1128/CMR.00030-19 PMC8262804 34043457

[5] Esposito A, Pompilio A, Bettua C, Crocetta V, Giacobazzi E, Fiscarelli E, Jousson O, Di Bonaventura G. 2017. Evolution of *Stenotrophomonas maltophilia* in cystic fibrosis lung over chronic infection: a genomic and phenotypic population study. Front Microbiol 8:1590. doi:10.3389/fmicb.2017.01590 PMC5581383 28894437

[6] Garazi M, Singer C, Tai J, Ginocchio CC. 2012. Bloodstream infections caused by *Stenotrophomonas maltophilia:* a seven-year review. J Hosp Infect 81:114–118. doi:10.1016/j.jhin.2012.02.008 22494851

[7] Boktour M, Hanna H, Ansari S, Bahna B, Hachem R, Tarrand J, Rolston K, Safdar A, Raad I. 2006. Central venous catheter and *Stenotrophomonas maltophilia* bacteremia in cancer patients. Cancer 106:1967–1973. doi:10.1002/cncr.21846 16565968

[8] Paez JIG, Costa SF. 2008. Risk factors associated with mortality of infections caused by *Stenotrophomonas maltophilia*: a systematic review. J Hosp Infect 70:101–108. doi:10.1016/j.jhin.2008.05.020 18621440

[9] Li K, Yu K, Huang Z, Liu X, Mei L, Ren X, Bai X, Gao H, Sun Z, Liu X, Wang D. 2024. *Stenotrophomonas maltophilia* complex: insights into evolutionary relationships, global distribution and pathogenicity. Front Cell Infect Microbiol 13:1325379. doi:10.3389/fcimb.2023.1325379 PMC10806987 38268792

[10] Gao S, Jin W, Quan Y, Li Y, Shen Y, Yuan S, Yi L, Wang Y, Wang Y. 2024. Bacterial capsules: occurrence, mechanism, and function. NPJ Biofilms Microbiomes 10:21. doi:10.1038/s41522-024-00497-6 PMC10937973 38480745

[11] Rendueles O, Garcia-Garcerà M, Néron B, Touchon M, Rocha EPC. 2017. Abundance and co-occurrence of extracellular capsules increase environmental breadth: implications for the emergence of pathogens. PLoS Pathog 13:e1006525. doi:10.1371/journal.ppat.1006525 PMC5542703 28742161

[12] Yother J. 2011. Capsules of *Streptococcus pneumoniae* and other bacteria: paradigms for polysaccharide biosynthesis and regulation. Annu Rev Microbiol 65:563–581. doi:10.1146/annurev.micro.62.081307.162944 21721938

[13] Kuipers A, Stapels DAC, Weerwind LT, Ko Y-P, Ruyken M, Lee JC, van Kessel KPM, Rooijakkers SHM. 2016. The *Staphylococcus aureus* polysaccharide capsule and Efb-dependent fibrinogen shield act in concert to protect against phagocytosis. Microbiology (Reading) 162:1185–1194. doi:10.1099/mic.0.000293 PMC4977062 27112346

[14] Campos MA, Vargas MA, Regueiro V, Llompart CM, Albertí S, Bengoechea JA. 2004. Capsule polysaccharide mediates bacterial resistance to antimicrobial peptides. Infect Immun 72:7107–7114. doi:10.1128/IAI.72.12.7107-7114.2004 PMC529140 15557634

[15] Cress BF, Englaender JA, He W, Kasper D, Linhardt RJ, Koffas MAG. 2014. Masquerading microbial pathogens: capsular polysaccharides mimic host-tissue molecules. FEMS Microbiol Rev 38:660–697. doi:10.1111/1574-6976.12056 PMC4120193 24372337

[16] Merino S, Tomás J. 2010. Bacterial capsules and evasion of immune responses. John Wiley & Sons, Ltd. https://onlinelibrary.wiley.com/doi/abs/10.1002/9780470015902.a0000957.pub3.

[17] Geisinger E, Isberg RR. 2015. Antibiotic modulation of capsular exopolysaccharide and virulence in *Acinetobacter baumannii**.* PLoS Pathog 11:e1004691. doi:10.1371/journal.ppat.1004691 PMC4334535 25679516

[18] Béchon N, Mihajlovic J, Vendrell-Fernández S, Chain F, Langella P, Beloin C, Ghigo J-M. 2020. Capsular polysaccharide cross-regulation modulates *Bacteroides thetaiotaomicron* biofilm formation. mBio 11:e00729-20. doi:10.1128/mBio.00729-20 PMC7315117 32576670

[19] Schembri MA, Dalsgaard D, Klemm P. 2004. Capsule shields the function of short bacterial adhesins. J Bacteriol 186:1249–1257. doi:10.1128/JB.186.5.1249-1257.2004 PMC344426 14973035

[20] Klimkaitė L, Armalytė J, Skerniškytė J, Sužiedėlienė E. 2020. The toxin-antitoxin systems of the opportunistic pathogen *Stenotrophomonas maltophilia* of environmental and clinical origin. Toxins (Basel) 12:635. doi:10.3390/toxins12100635 PMC7650669 33019620

[21] Wu JW, Wang JT, Lin TL, Liu YZ, Wu LT, Pan YJ. 2023. Identification of three capsule depolymerases in a bacteriophage infecting *Klebsiella pneumoniae* capsular types K7, K20, and K27 and therapeutic application. J Biomed Sci 30:31. doi:10.1186/s12929-023-00928-0 PMC10199534 37210493

[22] Cai R, Wang G, Le S, Wu M, Cheng M, Guo Z, Ji Y, Xi H, Zhao C, Wang X, Xue Y, Wang Z, Zhang H, Fu Y, Sun C, Feng X, Lei L, Yang Y, Ur Rahman S, Liu X, Han W, Gu J. 2019. Three capsular polysaccharide synthesis-related glucosyltransferases, GT-1, GT-2 and WcaJ, are associated with virulence and phage sensitivity of *Klebsiella pneumoniae*. Front Microbiol 10:1189. doi:10.3389/fmicb.2019.01189 PMC6546894 31191500

[23] Klimkaite L, Kukanauskaite G, Naujalis J, Ivanauskas D, Grigorjevas D, Petrenaite G, Skinkyte M, Porchun J, Zalyte E, Buchovec I, Skerniskyte J. 2026. Capsular polysaccharides of *Acinetobacter baumannii* modulate antimicrobial resistance and innate immune response. Sci Rep 16:14478. doi:10.1038/s41598-026-44001-w PMC13150009 41865055

[24] Tan YH, Chen Y, Chu WHW, Sham LT, Gan YH. 2020. Cell envelope defects of different capsule-null mutants in K1 hypervirulent *Klebsiella pneumoniae* can affect bacterial pathogenesis. Mol Microbiol 113:889–905. doi:10.1111/mmi.14447 PMC7317392 31912541

[25] Brown SP, Cornforth DM, Mideo N. 2012. Evolution of virulence in opportunistic pathogens: generalism, plasticity, and control. Trends Microbiol 20:336–342. doi:10.1016/j.tim.2012.04.005 PMC3491314 22564248

[26] Chauviat A, Abrouk D, Brothier E, Muller D, Meyer T, Favre-Bonté S. 2025. Genomic and phylogenetic re-assessment of the genus *Stenotrophomonas*: description of *Stenotrophomonas thermophila* sp. nov., and the proposal of *Parastenotrophomonas gen.* nov., *Pseudostenotrophomonas gen.* Nov., *Pedostenotrophomonas gen.* nov., and *Allostenotrophomonas gen.* nov. Syst Appl Microbiol 48:126630. doi:10.1016/j.syapm.2025.126630 40550196

[27] Mostowy RJ, Croucher NJ, De Maio N, Chewapreecha C, Salter SJ, Turner P, Aanensen DM, Bentley SD, Didelot X, Fraser C. 2017. Pneumococcal capsule synthesis locus cps as evolutionary hotspot with potential to generate novel serotypes by recombination. Mol Biol Evol 34:2537–2554. doi:10.1093/molbev/msx173 PMC5850285 28595308

[28] Larson MR, Biddle K, Gorman A, Boutom S, Rosenshine I, Saper MA. 2021. *Escherichia coli* O127 group 4 capsule proteins assemble at the outer membrane. PLoS One 16:e0259900. doi:10.1371/journal.pone.0259900 PMC8592465 34780538

[29] Hyams C, Camberlein E, Cohen JM, Bax K, Brown JS. 2010. The *Streptococcus pneumoniae* capsule inhibits complement activity and neutrophil phagocytosis by multiple mechanisms. Infect Immun 78:704–715. doi:10.1128/IAI.00881-09 PMC2812187 19948837

[30] Domenico P, Salo RJ, Cross AS, Cunha BA. 1994. Polysaccharide capsule-mediated resistance to opsonophagocytosis in *Klebsiella pneumoniae*. Infect Immun 62:4495–4499. doi:10.1128/iai.62.10.4495-4499.1994 PMC303135 7927714

[31] O’Riordan K, Lee JC. 2004. *Staphylococcus aureus* capsular polysaccharides. Clin Microbiol Rev 17:218–234. doi:10.1128/CMR.17.1.218-234.2004 PMC321462 14726462

[32] Looney WJ, Narita M, Mühlemann K. 2009. *Stenotrophomonas maltophilia*: an emerging opportunist human pathogen. Lancet Infect Dis 9:312–323. doi:10.1016/S1473-3099(09)70083-0 19393961

[33] Di Bonaventura G, Spedicato I, D’Antonio D, Robuffo I, Piccolomini R. 2004. Biofilm formation by *Stenotrophomonas maltophilia*: modulation by quinolones, trimethoprim-sulfamethoxazole, and ceftazidime. Antimicrob Agents Chemother 48:151–160. doi:10.1128/AAC.48.1.151-160.2004 PMC310151 14693533

[34] Singh JK, Adams FG, Brown MH. 2018. Diversity and function of capsular polysaccharide in *Acinetobacter baumannii*. Front Microbiol 9:3301. doi:10.3389/fmicb.2018.03301 PMC6333632 30687280

[35] Skerniškytė J, Krasauskas R, Péchoux C, Kulakauskas S, Armalytė J, Sužiedėlienė E. 2019. Surface-related features and virulence among *Acinetobacter baumannii* clinical isolates belonging to international clones I and II. Front Microbiol 9. doi:10.3389/fmicb.2018.03116 PMC6331429 30671029

[36] Tipton KA, Chin CY, Farokhyfar M, Weiss DS, Rather PN. 2018. Role of capsule in resistance to disinfectants, host antimicrobials, and desiccation in *Acinetobacter baumannii*. Antimicrob Agents Chemother 62:e01188-18. doi:10.1128/AAC.01188-18 PMC6256795 30297362

[37] Gil-Gil T, Martínez JL, Blanco P. 2020. Mechanisms of antimicrobial resistance in *Stenotrophomonas maltophilia*: a review of current knowledge. Expert Rev Anti Infect Ther 18:335–347. doi:10.1080/14787210.2020.1730178 32052662

[38] Thurlow CM, Hossain MJ, Sun D, Barger P, Foshee L, Beck BH, Newton JC, Terhune JS, Saper MA, Liles MR. 2019. The gfc operon is involved in the formation of the O antigen capsule in *Aeromonas hydrophila* and contributes to virulence in channel catfish. Aquaculture 512:734334. doi:10.1016/j.aquaculture.2019.734334

[39] Peleg A, Shifrin Y, Ilan O, Nadler-Yona C, Nov S, Koby S, Baruch K, Altuvia S, Elgrably-Weiss M, Abe CM, Knutton S, Saper MA, Rosenshine I. 2005. Identification of an *Escherichia coli* operon required for formation of the O-antigen capsule. J Bacteriol 187:5259–5266. doi:10.1128/JB.187.15.5259-5266.2005 PMC1196049 16030220

[40] Nadler C, Koby S, Peleg A, Johnson AC, Suddala KC, Sathiyamoorthy K, et al.. 2012. Cycling of Etk and Etp phosphorylation states is involved in formation of group 4 capsule by *Escherichia coli**.* PLoS One 7:e37984. doi:10.1371/journal.pone.0037984 PMC3366997 22675501

[41] Hanson BR, Runft DL, Streeter C, Kumar A, Carion TW, Neely MN. 2012. Functional analysis of the CpsA protein of *Streptococcus agalactiae*. J Bacteriol 194:1668–1678. doi:10.1128/JB.06373-11 PMC3302465 22287515

[42] Locke JB, Colvin KM, Datta AK, Patel SK, Naidu NN, Neely MN, Nizet V, Buchanan JT. 2007. *Streptococcus iniae* capsule impairs phagocytic clearance and contributes to virulence in fish. J Bacteriol 189:1279–1287. doi:10.1128/JB.01175-06 PMC1797360 17098893

[43] Kalynych S, Morona R, Cygler M. 2014. Progress in understanding the assembly process of bacterial O-antigen. FEMS Microbiol Rev 38:1048–1065. doi:10.1111/1574-6976.12070 24617522

[44] Williams KP. 2002. Integration sites for genetic elements in prokaryotic tRNA and tmRNA genes: sublocation preference of integrase subfamilies. Nucleic Acids Res 30:866–875. doi:10.1093/nar/30.4.866 PMC100330 11842097

[45] Juhas M, van der Meer JR, Gaillard M, Harding RM, Hood DW, Crook DW. 2009. Genomic islands: tools of bacterial horizontal gene transfer and evolution. FEMS Microbiol Rev 33:376–393. doi:10.1111/j.1574-6976.2008.00136.x PMC2704930 19178566

[46] Sun Q, Knirel YA, Wang J, Luo X, Senchenkova SN, Lan R, Shashkov AS, Xu J. 2014. Serotype-converting bacteriophage SfII encodes an acyltransferase protein that mediates 6-O-acetylation of GlcNAc in *Shigella flexneri* O-antigens, conferring on the host a novel O-antigen epitope. J Bacteriol 196:3656–3666. doi:10.1128/JB.02009-14 PMC4187690 25112477

[47] Rendueles O, de Sousa JAM, Bernheim A, Touchon M, Rocha EPC. 2018. Genetic exchanges are more frequent in bacteria encoding capsules. Genet 14:e1007862. doi:10.1371/journal.pgen.1007862 PMC6322790 30576310

[48] Hassan F, Türkyılmaz S, El-Mahdy TS. 2026. Genomic analysis identifies candidate SOS-associated and EPS/envelope-remodeling islands in a drinking-water *Stenotrophomonas maltophilia* complex isolate. Sci Rep 16:19640. doi:10.1038/s41598-026-58581-0 PMC13310844 42366209

[49] Walsh RL, Camilli A. 2011. *Streptococcus pneumoniae* is desiccation tolerant and infectious upon rehydration. mBio 2:e00092-11. doi:10.1128/mBio.00092-11 PMC3101785 21610120

[50] Wang H, Shen J, Ma K, Zhu C, Fang M, Hou X, Zhang S, Wang W, Xue T. 2022. Transcriptome analysis revealed the role of capsular polysaccharides in desiccation tolerance of foodborne *Staphylococcus aureus*. Food Res Int 159:111602. doi:10.1016/j.foodres.2022.111602 35940797

[51] Pompilio A, Crocetta V, Confalone P, Nicoletti M, Petrucca A, Guarnieri S, Fiscarelli E, Savini V, Piccolomini R, Di Bonaventura G. 2010. Adhesion to and biofilm formation on IB3-1 bronchial cells by *Stenotrophomonas maltophilia* isolates from cystic fibrosis patients. BMC Microbiol 10:102. doi:10.1186/1471-2180-10-102 PMC2858031 20374629

[52] de Oliveira-Garcia D, Dall’Agnol M, Rosales M, Azzuz ACGS, Alcántara N, Martinez MB, Girón JA. 2003. Fimbriae and adherence of *Stenotrophomonas maltophilia* to epithelial cells and to abiotic surfaces. Cell Microbiol 5:625–636. doi:10.1046/j.1462-5822.2003.00306.x 12925132

[53] Schembri MA, Blom J, Krogfelt KA, Klemm P. 2005. Capsule and fimbria interaction in *Klebsiella pneumoniae*. Infect Immun 73:4626–4633. doi:10.1128/IAI.73.8.4626-4633.2005 PMC1201234 16040975

[54] Doorduijn DJ, Rooijakkers SHM, van Schaik W, Bardoel BW. 2016. Complement resistance mechanisms of *Klebsiella pneumoniae*. Immunobiology 221:1102–1109. doi:10.1016/j.imbio.2016.06.014 27364766

[55] Hsieh SA, Allen PM. 2020. Immunomodulatory roles of polysaccharide capsules in the intestine. Front Immunol 11:690. doi:10.3389/fimmu.2020.00690 PMC7174666 32351514

[56] Zhang LF, Lepenies B, Nakamae S, Young BM, Santos RL, Raffatellu M, Cobb BA, Hiyoshi H, Bäumler AJ. 2022. The Vi capsular polysaccharide of *Salmonella typhi* promotes macrophage phagocytosis by binding the human C-type lectin DC-SIGN. mBio 13:e02733-22. doi:10.1128/mbio.02733-22 PMC9765441 36286551

[57] Jiang Z, Song F, Li Y, Xue D, Deng G, Li M, Liu X, Wang Y. 2017. Capsular polysaccharide is a main component of *Mycoplasma ovipneumoniae* in the pathogen-induced toll-like receptor-mediated inflammatory responses in sheep airway epithelial cells. Mediators Inflamm 2017:9891673. doi:10.1155/2017/9891673 PMC5434471 28553017

[58] Härtel C, Scholz T, Kuhn M, Bendiks M, Göpel W, Lauten M, Herting E. 2013. Innate immune responses to *Stenotrophomonas maltophilia* in immunocompromised pediatric patients and the effect of taurolidine. J Microbiol Immunol Infect 46:115–120. doi:10.1016/j.jmii.2012.04.002 22727544

[59] Kim YJ, Jeon H, Na SH, Kwon HI, Selasi GN, Nicholas A, Park TI, Lee SH, Lee JC. 2016. *Stenotrophomonas maltophilia* outer membrane vesicles elicit a potent inflammatory response *in vitro* and *in vivo**.* Pathog Dis 74:ftw104. doi:10.1093/femspd/ftw104 27756813

[60] Zgair AK, Al-Adressi AMH. 2013. *Stenotrophomonas maltophilia* fimbrin stimulates mouse bladder innate immune response. Eur J Clin Microbiol Infect Dis 32:139–146. doi:10.1007/s10096-012-1729-0 22910808

[61] Di Bonaventura G, Pompilio A, Zappacosta R, Petrucci F, Fiscarelli E, Rossi C, Piccolomini R. 2010. Role of excessive inflammatory response to *Stenotrophomonas maltophilia* lung infection in DBA/2 mice and implications for cystic fibrosis. Infect Immun 78:2466–2476. doi:10.1128/IAI.01391-09 PMC2876550 20308302

[62] Sawant KV, Sepuru KM, Lowry E, Penaranda B, Frevert CW, Garofalo RP, Rajarathnam K. 2021. Neutrophil recruitment by chemokines Cxcl1/KC and Cxcl2/MIP2: Role of Cxcr2 activation and glycosaminoglycan interactions. J Leukoc Biol 109:777–791. doi:10.1002/JLB.3A0820-207R PMC8296306 32881070

[63] Peñaloza HF, Schultz BM, Nieto PA, Salazar GA, Suazo I, Gonzalez PA, Riedel CA, Alvarez-Lobos MM, Kalergis AM, Bueno SM. 2016. Opposing roles of IL-10 in acute bacterial infection. Cytokine Growth Factor Rev 32:17–30. doi:10.1016/j.cytogfr.2016.07.003 27522641

[64] Jansen AM, Hall LJ, Clare S, Goulding D, Holt KE, Grant AJ, Mastroeni P, Dougan G, Kingsley RA. 2011. A *Salmonella* Typhimurium-Typhi genomic chimera: a model to study Vi polysaccharide capsule function *in vivo*. PLoS Pathog 7:e1002131. doi:10.1371/journal.ppat.1002131 PMC3145788 21829346

[65] Küng E, Coward WR, Neill DR, Malak HA, Mühlemann K, Kadioglu A, Hilty M, Hathaway LJ. 2014. The *Pneumococcal polysaccharide* capsule and pneumolysin differentially affect CXCL8 and IL-6 release from cells of the upper and lower respiratory tract. PLoS One 9:e92355. doi:10.1371/journal.pone.0092355 PMC3963895 24664110

[66] Klimkaitė L, Drevinskaitė R, Krinickis K, Sužiedėlienė E, Armalytė J. 2025. *Stenotrophomonas maltophilia* of clinical origin display higher temperature tolerance comparing with environmental isolates. Virulence 16:2498669. doi:10.1080/21505594.2025.2498669 PMC12064055 40314203

[67] Yanisch-Perron C, Vieira J, Messing J. 1985. Improved M13 phage cloning vectors and host strains: nucleotide sequences of the M13mp18 and pUC19 vectors. Gene 33:103–119. doi:10.1016/0378-1119(85)90120-9 2985470

[68] Krasauskas R, Skerniškytė J, Armalytė J, Sužiedėlienė E. 2019. The role of *Acinetobacter baumannii* response regulator BfmR in pellicle formation and competitiveness via contact-dependent inhibition system. BMC Microbiol 19:241. doi:10.1186/s12866-019-1621-5 PMC6833216 31690263

[69] Ye X, Dong H, Huang YP. 2014. Highly efficient transformation of *Stenotrophomonas maltophilia* S21, an environmental isolate from soil, by electroporation. J Microbiol Methods 107:92–97. doi:10.1016/j.mimet.2014.09.010 25300664

[70] Dorman MJ, Feltwell T, Goulding DA, Parkhill J, Short FL. 2018. The capsule regulatory network of *Klebsiella pneumoniae* defined by density-TraDISort. mBio 9:e01863-18. doi:10.1128/mBio.01863-18 PMC6247091 30459193

[71] Birkhead M, Ganesh K, Ndlangisa KM, Koornhof HJ. 2017. Transmission electron microscopy protocols forcapsule visualisation in pathogenic respiratory and meningeal bacteria, p 628–639. In Mendez-Vilas A (ed), Microscopy and imaging science: practical approaches to applied research and education. Formatex Research Centre, Badajoz, Spain.

[72] Snyderman R, Pike MC, Fischer DG, Koren HS. 1977. Biologic and biochemical activities of continuous macrophage cell lines P388D1 and J774.1. J Immunol 119:2060–2066. doi:10.4049/jimmunol.119.6.2060 915291

[73] Lieber M, Smith B, Szakal A, Nelson-Rees W, Todaro G. 1976. A continuous tumor-cell line from a human lung carcinoma with properties of type II alveolar epithelial cells. Int J Cancer 17:62–70. doi:10.1002/ijc.2910170110 175022

[74] Livak KJ, Schmittgen TD. 2001. Analysis of relative gene expression data using real-time quantitative PCR and the 2^−ΔΔ^*^C^*^_T_^ method. Methods 25:402–408. doi:10.1006/meth.2001.1262 11846609

[75] Wick RR, Howden BP, Stinear TP. 2025. Autocycler: long-read consensus assembly for bacterial genomes. Bioinformatics 41:btaf474. doi:10.1093/bioinformatics/btaf474 PMC12460055 40875535

[76] Koren S, Walenz BP, Berlin K, Miller JR, Bergman NH, Phillippy AM. 2017. Canu: scalable and accurate long-read assembly via adaptive *k*-mer weighting and repeat separation. Genome Res 27:722–736. doi:10.1101/gr.215087.116 PMC5411767 28298431

[77] Kolmogorov M, Yuan J, Lin Y, Pevzner PA. 2019. Assembly of long, error-prone reads using repeat graphs. Nat Biotechnol 37:540–546. doi:10.1038/s41587-019-0072-8 30936562

[78] Benoit G, Raguideau S, James R, Phillippy AM, Chikhi R, Quince C. 2024. High-quality metagenome assembly from long accurate reads with metaMDBG. Nat Biotechnol 42:1378–1383. doi:10.1038/s41587-023-01983-6 PMC11392814 38168989

[79] Vaser R, Šikić M. 2021. Time- and memory-efficient genome assembly with Raven. Nat Comput Sci 1:332–336. doi:10.1038/s43588-021-00073-4 38217213

[80] Li H. 2016. Minimap and miniasm: fast mapping and *de novo* assembly for noisy long sequences. Bioinformatics 32:2103–2110. doi:10.1093/bioinformatics/btw152 PMC4937194 27153593

[81] Chen Y, Nie F, Xie S-Q, Zheng Y-F, Dai Q, Bray T, Wang Y-X, Xing J-F, Huang Z-J, Wang D-P, He L-J, Luo F, Wang J-X, Liu Y-Z, Xiao C-L. 2021. Efficient assembly of nanopore reads via highly accurate and intact error correction. Nat Commun 12:60. doi:10.1038/s41467-020-20236-7 PMC7782737 33397900

[82] Hu J, Wang Z, Sun Z, Hu B, Ayoola AO, Liang F, Li J, Sandoval JR, Cooper DN, Ye K, Ruan J, Xiao C-L, Wang D, Wu D-D, Wang S. 2024. NextDenovo: an efficient error correction and accurate assembly tool for noisy long reads. Genome Biol 25:107. doi:10.1186/s13059-024-03252-4 PMC11046930 38671502

[83] Ruan J, Li H. 2020. Fast and accurate long-read assembly with wtdbg2. Nat Methods 17:155–158. doi:10.1038/s41592-019-0669-3 PMC7004874 31819265

[84] Bouras G, Grigson SR, Papudeshi B, Mallawaarachchi V, Roach MJ. 2024. Dnaapler: a tool to reorient circular microbial genomes. JOSS 9:5968. doi:10.21105/joss.05968

[85] Schwengers O, Jelonek L, Dieckmann MA, Beyvers S, Blom J, Goesmann A. 2021. Bakta: rapid and standardized annotation of bacterial genomes via alignment-free sequence identification. Microb Genom 7. doi:10.1099/mgen.0.000685 PMC8743544 34739369

[86] Chaumeil PA, Mussig AJ, Hugenholtz P, Parks DH. 2022. GTDB-Tk v2: memory friendly classification with the genome taxonomy database. Bioinformatics 38:5315–5316. doi:10.1093/bioinformatics/btac672 PMC9710552 36218463

[87] Skani enables accurate and efficient genome comparison for modern metagenomic datasets. 2023. Nat Methods 20:1633–1634. doi:10.1038/s41592-023-02019-2 37735571

[88] Bu C, Zhang H, Zhang F, Liang W, Gao H, Zhao J, Lv F, Xue R, Liu Q, Zhang Z, Jin Z, Xiao J. 2025. PGAP2: a comprehensive toolkit for prokaryotic pan-genome analysis based on fine-grained feature networks. Nat Commun 16:9865. doi:10.1038/s41467-025-64846-5 PMC12603064 41213900

[89] Zhou S, Liu B, Zheng D, Chen L, Yang J. 2025. VFDB 2025: an integrated resource for exploring anti-virulence compounds. Nucleic Acids Res 53:D871–D877. doi:10.1093/nar/gkae968 PMC11701737 39470738

[90] Varadi M, Bertoni D, Magana P, Paramval U, Pidruchna I, Radhakrishnan M, Tsenkov M, Nair S, Mirdita M, Yeo J, Kovalevskiy O, Tunyasuvunakool K, Laydon A, Žídek A, Tomlinson H, Hariharan D, Abrahamson J, Green T, Jumper J, Birney E, Steinegger M, Hassabis D, Velankar S. 2024. AlphaFold protein structure database in 2024: providing structure coverage for over 214 million protein sequences. Nucleic Acids Res 52:D368–D375. doi:10.1093/nar/gkad1011 PMC10767828 37933859

[91] van Kempen M, Kim SS, Tumescheit C, Mirdita M, Lee J, Gilchrist CLM, Söding J, Steinegger M. 2024. Fast and accurate protein structure search with Foldseek. Nat Biotechnol 42:243–246. doi:10.1038/s41587-023-01773-0 PMC10869269 37156916

[92] Bertoni D, Tsenkov M, Magana P, Nair S, Pidruchna I, Querino Lima Afonso M, Midlik A, Paramval U, Lawal D, Tanweer A, et al.. 2026. AlphaFold protein structure database 2025: a redesigned interface and updated structural coverage. Nucleic Acids Res 54:D358–D362. doi:10.1093/nar/gkaf1226 PMC12807749 41273079

[93] Kim RS, Levy Karin E, Mirdita M, Chikhi R, Steinegger M. 2025. BFVD-a large repository of predicted viral protein structures. Nucleic Acids Res 53:D340–D347. doi:10.1093/nar/gkae1119 PMC11701548 39574394

