# Supplementary material for: The role of the polysaccharide capsule as a novel virulence-associated trait of Stenotrophomonas maltophilia complex bacteria
Source: mBio. 2026 Sep 11;17(10):e01635-26. doi: 10.1128/mbio.01635-26 (PMC13712831; doi:10.1128/mbio.01635-26)
Supplement: Supplementary file 1 [file mbio.01635-26-s0002.pdf]

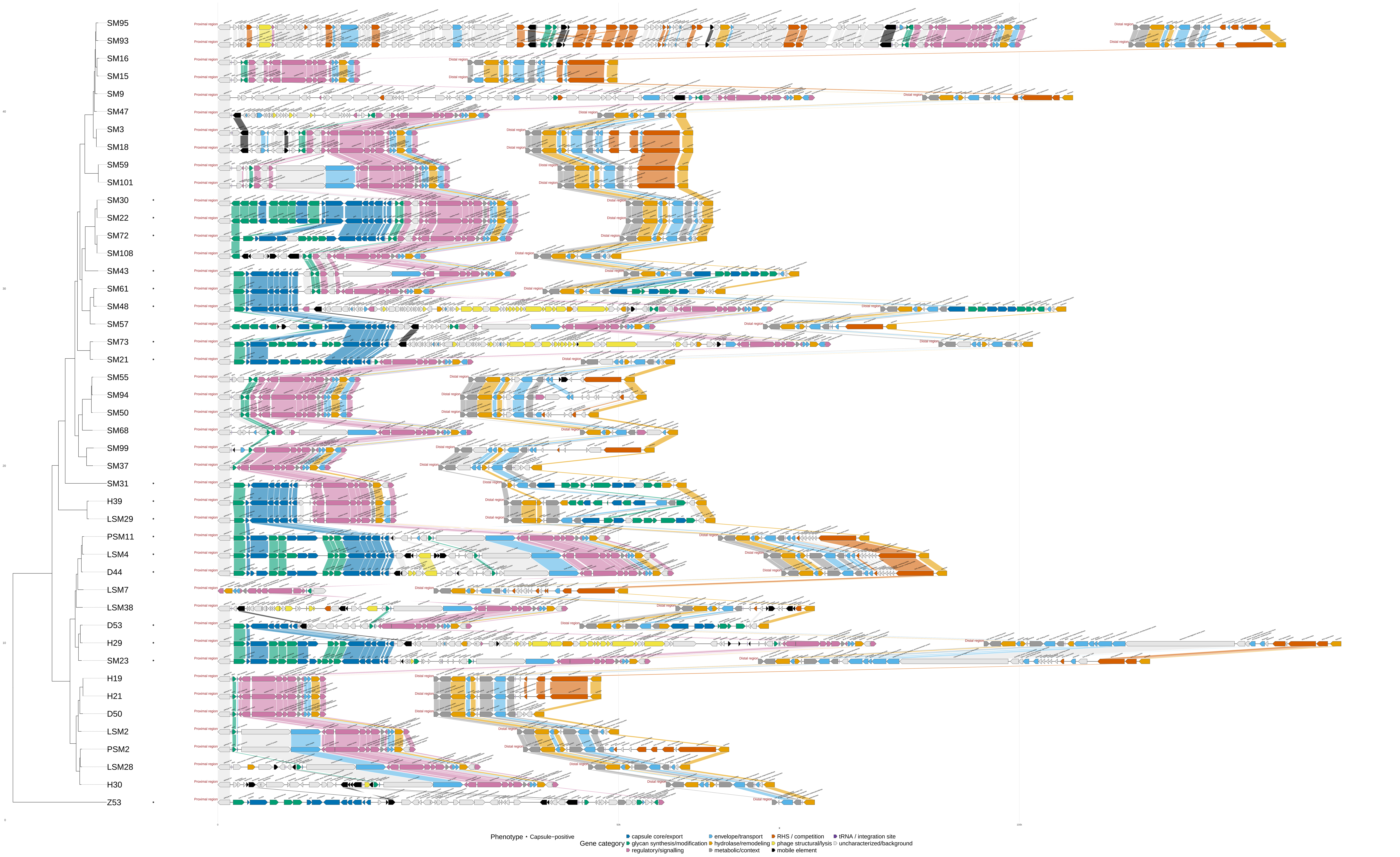
SM95
SM93
SM16
SM15
SM9
SM47
SM3
SM18
SM59
SM101
SM30
SM22
SM72
SM108
SM43
SM61
SM48
SM57
SM73
SM21
SM55
SM94
SM50
SM68
SM99
SM37
SM31
H39
LSM29
PSM11
LSM4
D44
LSM7
LSM38
D53
H29
SM23
H19
H21
D50
LSM2
PSM2
LSM28
H30
Z53
Proximal region
Distal region
Phenotype • Capsule−positive
Gene category
capsule core/export
glycan synthesis/modification
regulatory/signalling
envelope/transport
hydrolase/remodeling
metabolic/context
RHS / competition
phage structural/lysis
mobile element
tRNA / integration site
uncharacterized/background
