# Supplementary material for: The role of the polysaccharide capsule as a novel virulence-associated trait of Stenotrophomonas maltophilia complex bacteria
Source: mBio. 2026 Sep 11;17(10):e01635-26. doi: 10.1128/mbio.01635-26 (PMC13712831; doi:10.1128/mbio.01635-26)
Supplement: Supplementary file 2 [file mbio.01635-26-s0003.pdf]

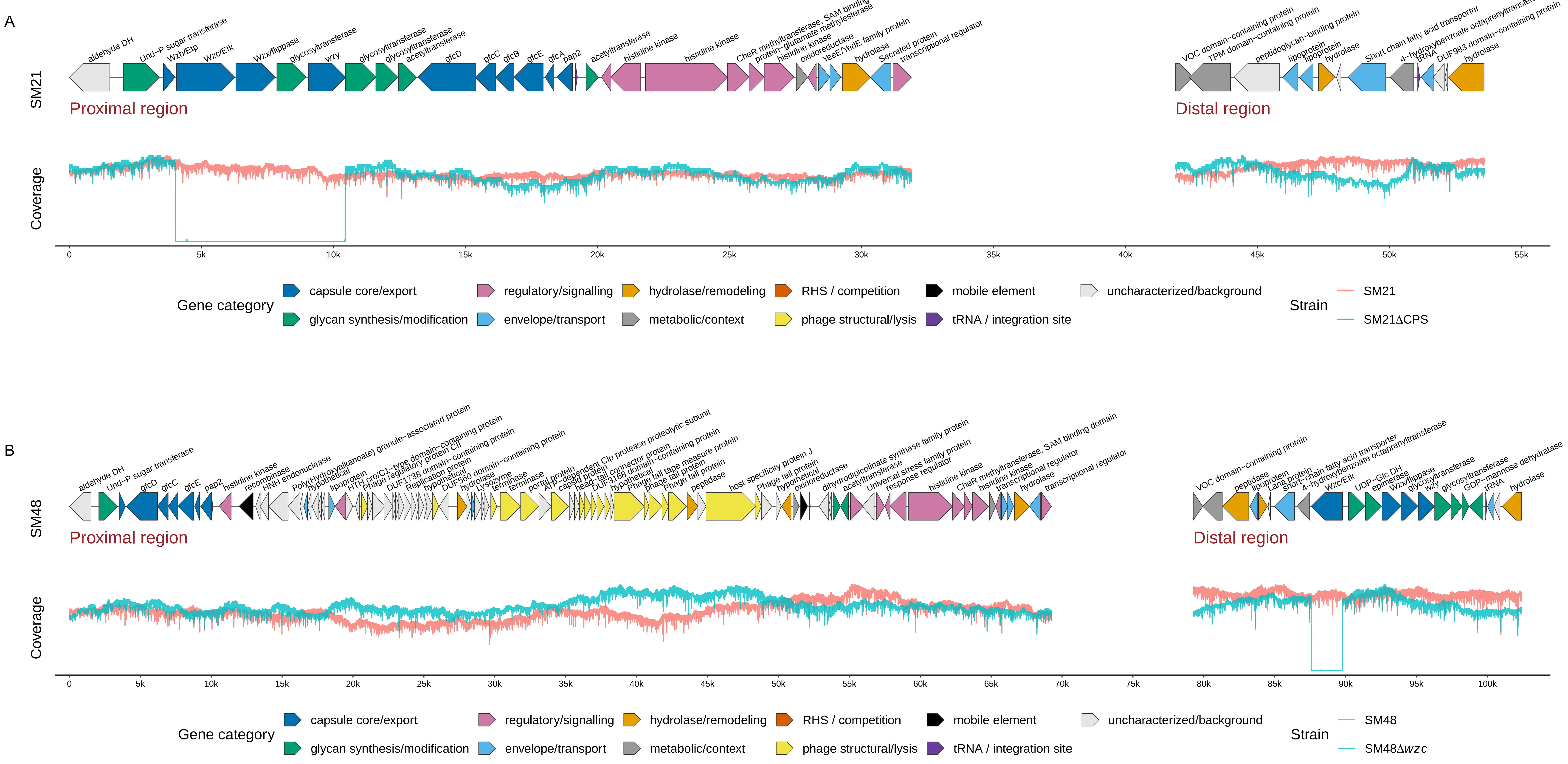
A
SM21
aldehyde DH
Und–P sugar transferase
Wzb/Etp
Wzc/Etk
Wzx/flippase
glycosyltransferase
wzy
glycosyltransferase
glycosyltransferase
acetyltransferase
gfcD
gfcC
gfcB
gfcE
gfcA
pap2
acetyltransferase
histidine kinase
histidine kinase
CheR methyltransferase, SAM binding
protein–glutamate methylesterase
histidine kinase
oxidoreductase
YeeE/YedE family protein
hydrolase
Secreted protein
transcriptional regulator
VOC domain–containing protein
TPM domain–containing protein
peptidoglycan–binding protein
lipoprotein
lipoprotein
hydrolase
Short chain fatty acid transporter
4–hydroxybenzoate octaprenyltransferase
tRNA
DUF983 domain–containing protein
hydrolase
Proximal region
Distal region
Coverage
0
5k
10k
15k
20k
25k
30k
35k
40k
45k
50k
55k
Gene category
capsule core/export
glycan synthesis/modification
regulatory/signalling
envelope/transport
hydrolase/remodeling
metabolic/context
RHS / competition
phage structural/lysis
mobile element
tRNA / integration site
uncharacterized/background
Strain
SM21
SM21ΔCPS
B
SM48
aldehyde DH
Und–P sugar transferase
gfcD
gfcC
gfcE
pap2
histidine kinase
recombinase
HNH endonuclease
Poly(Hydroxyalkanoate) granule–associated protein
hypothetical
lipoprotein
HTH cro/C1–type domain–containing protein
Phage regulatory protein CII
DUF1738 domain–containing protein
Replication protein
hypothetical
DUF560 domain–containing protein
hydrolase
Lysozyme
terminase
terminase
portal protein
ATP–dependent Clp protease proteolytic subunit
capsid protein
head–tail connector protein
DUF3168 domain–containing protein
hypothetical
Phage tail tape measure protein
phage tail protein
Phage tail protein
peptidase
host specificity protein J
Phage tail protein
hypothetical
oxidoreductase
dihydrodipicolinate synthase family protein
acetyltransferase
Universal stress family protein
response regulator
histidine kinase
CheR methyltransferase, SAM binding domain
histidine kinase
transcriptional regulator
hydrolase
transcriptional regulator
VOC domain–containing protein
peptidase
lipoprotein
Lana protein
Short–chain fatty acid transporter
4–hydroxybenzoate octaprenyltransferase
Wzc/Etk
UDP–Glc DH
epimerase
Wzx/flippase
glycosyltransferase
wzy
glycosyltransferase
GDP–mannose dehydratase
tRNA
hydrolase
Proximal region
Distal region
Coverage
0
5k
10k
15k
20k
25k
30k
35k
40k
45k
50k
55k
60k
65k
70k
75k
80k
85k
90k
95k
100k
Gene category
capsule core/export
glycan synthesis/modification
regulatory/signalling
envelope/transport
hydrolase/remodeling
metabolic/context
RHS / competition
phage structural/lysis
mobile element
tRNA / integration site
uncharacterized/background
Strain
SM48
SM48Δwzc
