# Supplementary material for: The role of the polysaccharide capsule as a novel virulence-associated trait of Stenotrophomonas maltophilia complex bacteria
Source: mBio. 2026 Sep 11;17(10):e01635-26. doi: 10.1128/mbio.01635-26 (PMC13712831; doi:10.1128/mbio.01635-26)
Supplement: Supplementary file 3 [file mbio.01635-26-s0006.docx]

**Supplementary Figure legends:**

**Supplementary Fig. 1. Evaluation of the polysaccharide capsule phenotype in a collection of *S. maltophilia* complex isolates of environmental (A) and clinical (B) origin.** The upper panels show the results of density gradient fractionation; the lower panels show polysaccharide extracts separated by SDS-PAGE and stained with Alcian Blue. * indicates capsule-producing isolates.

**Supplementary Fig. 2. Organization of proximal and distal capsule-associated loci.** Genomic organization of proximal and distal capsule-associated loci across the analyzed *S. maltophilia* complex isolates. Genes are colored by functional category, and isolates are ordered according to the core-genome phylogeny. Capsule-positive isolates are indicated next to the tree. This supplementary figure provides broader locus-level detail for the capsule-associated regions summarized in Fig. 3.

**Supplementary Fig. 3.** **Scheme demonstrating the polysaccharide capsule synthesis-related genetic operons of clinical *S. maltophilia* complex isolates SM21 (A) and SM48 (B).** Coverage plots show aligned raw genome sequencing reads of capsulated isolates SM21 and SM48, along with their capsule-deficient mutants SM21ΔCPS and SM48Δ*wzc*, providing evidence of CPS synthesis-associated gene deletions.

**Supplementary Fig. 4.** **Analysis of polysaccharide capsule-expressing *S. maltophilia* complex isolates SM21 and SM48 and their capsule-deficient mutants.** (A) Polysaccharide capsule operon deletions identified by PCR method; (B) Polysaccharide capsule phenotype analysis by density-dependent gradient test; (C) Polysaccharide capsule phenotype analysis using Alcian Blue dye, white arrows indicate capsular polysaccharides; (D) Isolates growth in nutrient-rich LB medium.

**Supplementary Fig. 5.** **Visualization of clinical and environmental *S. maltophilia* complex isolates' polysaccharide capsule using TEM, with optimized sample preparation technique.** Representative images of capsulated clinical and environmental *S. maltophilia* isolates (A) and non-capsulated clinical and environmental *S. maltophilia* isolates (B). White arrows indicate the polysaccharide capsule.
