# Supplementary material for: The role of the polysaccharide capsule as a novel virulence-associated trait of Stenotrophomonas maltophilia complex bacteria
Source: mBio. 2026 Sep 11;17(10):e01635-26. doi: 10.1128/mbio.01635-26 (PMC13712831; doi:10.1128/mbio.01635-26)
Supplement: Supplementary file 4 [file mbio.01635-26-s0010.docx]

**Supplementary Table 4.** Clinical and environmental *S. maltophilia* complex isolates used in this study.

| ***Stenotrophomonas maltophilia* complex isolate** | **Origin** | **Source*** | **Year of isolation** | **Reference** |
| --- | --- | --- | --- | --- |
| D34 | Environmental | Soil | 2016 | (1) |
| D44 | Environmental | Soil | 2016 | (1) |
| D46 | Environmental | Soil | 2016 | (1) |
| D50 | Environmental | Soil | 2016 | (1) |
| D53 | Environmental | Soil | 2016 | (1) |
| D150 | Environmental | Soil | 2016 | (1) |
| Z53 | Environmental | Fish | 2016 | (1) |
| H4 | Environmental | Fish | 2020 | (2) |
| H11 | Environmental | Fish | 2020 | (2) |
| H19 | Environmental | Fish | 2020 | (2) |
| H20 | Environmental | Fish | 2020 | (2) |
| H21 | Environmental | Fish | 2020 | (2) |
| H26 | Environmental | Fish | 2020 | (2) |
| H29 | Environmental | Fish | 2020 | (2) |
| H30 | Environmental | Fish | 2020 | (2) |
| H39 | Environmental | Fish | 2020 | (2) |
| LSM1 | Environmental | Soil | 2021 | (2) |
| LSM2 | Environmental | Soil | 2021 | (2) |
| LSM4 | Environmental | Soil | 2021 | (2) |
| LSM5 | Environmental | Soil | 2021 | (2) |
| LSM6 | Environmental | Soil | 2021 | (2) |
| LSM7 | Environmental | Soil | 2021 | (2) |
| LSM8 | Environmental | Soil | 2021 | (2) |
| LSM10 | Environmental | Soil | 2021 | (2) |
| LSM19 | Environmental | Soil | 2021 | (2) |
| LSM23 | Environmental | Soil | 2021 | (2) |
| LSM25 | Environmental | Soil | 2021 | (2) |
| LSM26 | Environmental | Soil | 2021 | (2) |
| LSM27 | Environmental | Soil | 2021 | (2) |
| LSM28 | Environmental | Soil | 2021 | (2) |
| LSM29 | Environmental | Soil | 2021 | (2) |
| LSM30 | Environmental | Soil | 2021 | (2) |
| LSM31 | Environmental | Soil | 2021 | (2) |
| LSM32 | Environmental | Soil | 2021 | (2) |
| LSM33 | Environmental | Soil | 2021 | (2) |
| LSM38 | Environmental | Soil | 2021 | (2) |
| LSM39 | Environmental | Soil | 2021 | (2) |
| LSM40 | Environmental | Soil | 2021 | (2) |
| LSM42 | Environmental | Soil | 2021 | (2) |
| PSM2 | Environmental | Soil | 2024 | Acquired from I. Ragaišis |
| PSM11 | Environmental | Soil | 2024 | Acquired from I. Ragaišis |
| R551-3 | Environmental |  |  | (3) |
| SM3 | Clinical | NCI | 2017 | (1) |
| SM5 | Clinical | VCCH LM | 2018 | (1) |
| SM6 | Clinical | VCCH LM | 2018 | (1) |
| SM7 | Clinical | VCCH LM | 2018 | (1) |
| SM8 | Clinical | NCI | 2019 | (1) |
| SM9 | Clinical | NCI | 2019 | (1) |
| SM10 | Clinical | VCCH LM | 2019 | (1) |
| SM11 | Clinical | VUH SK PD | 2019 | (1) |
| SM12 | Clinical | VUH SK PD | 2019 | (1) |
| SM13 | Clinical | VUH SK PD | 2019 | (1) |
| SM14 | Clinical | VCCH LM | 2019 | (1) |
| SM15 | Clinical | KCPHC | 2019 | (1) |
| SM16 | Clinical | VCCH LM | 2019 | (1) |
| SM17 | Clinical | NCI | 2019 | (1) |
| SM18 | Clinical | NCI | 2019 | (1) |
| SM20 | Clinical | NCI | 2019 | (1) |
| SM21 | Clinical | NCI | 2019 | (1) |
| SM22 | Clinical | NCI | 2019 | (1) |
| SM23 | Clinical | NCI | 2019 | (1) |
| SM24 | Clinical | NCI | 2019 | (1) |
| SM25 | Clinical | NCI | 2019 | (1) |
| SM27 | Clinical | NCI | 2019 | (2) |
| SM28 | Clinical | VCCH LM | 2020 | (2) |
| SM29 | Clinical | VCCH LM | 2021 | (2) |
| SM30 | Clinical | VCCH LM | 2021 | (2) |
| SM31 | Clinical | VCCH LM | 2021 | (2) |
| SM32 | Clinical | VCCH LM | 2021 | (2) |
| SM33 | Clinical | VCCH LM | 2021 | (2) |
| SM34 | Clinical | VCCH LM | 2021 | (2) |
| SM35 | Clinical | VCCH LM | 2021 | (2) |
| SM36 | Clinical | VCCH LM | 2021 | (2) |
| SM37 | Clinical | VCCH LM | 2021 | (2) |
| SM38 | Clinical | VCCH LM | 2021 | (2) |
| SM39 | Clinical | VCCH LM | 2021 | (2) |
| D457 | Clinical |  | 2012 | (4) |
| SM40 | Clinical | VCCH LM | 2022 | This work |
| SM41 | Clinical | VLSK LMC ML | 2022 | This work |
| SM42 | Clinical | VULSK LMC ML | 2022 | This work |
| SM43 | Clinical | VULSK LMC ML | 2022 | This work |
| SM44 | Clinical | VULSK LMC ML | 2022 | This work |
| SM45 | Clinical | VULSK LMC ML | 2022 | This work |
| SM46 | Clinical | VULSK LMC ML | 2022 | This work |
| SM47 | Clinical | VULSK LMC ML | 2022 | This work |
| SM48 | Clinical | VULSK LMC ML | 2022 | This work |
| SM49 | Clinical | VULSK LMC ML | 2022 | This work |
| SM50 | Clinical | VULSK LMC ML | 2022 | This work |
| SM51 | Clinical | VULSK LMC ML | 2022 | This work |
| SM52 | Clinical | VULSK LMC ML | 2022 | This work |
| SM53 | Clinical | VULSK LMC ML | 2022 | This work |
| SM54 | Clinical | VULSK LMC ML | 2022 | This work |
| SM55 | Clinical | VULSK LMC ML | 2022 | This work |
| SM56 | Clinical | VULSK LMC ML | 2023 | This work |
| SM57 | Clinical | VULSK LMC ML | 2023 | This work |
| SM58 | Clinical | VULSK LMC ML | 2023 | This work |
| SM59 | Clinical | VULSK LMC ML | 2023 | This work |
| SM60 | Clinical | VULSK LMC ML | 2023 | This work |
| SM61 | Clinical | VULSK LMC ML | 2023 | This work |
| SM62 | Clinical | VULSK LMC ML | 2023 | This work |
| SM63 | Clinical | VULSK LMC ML | 2023 | This work |
| SM64 | Clinical | VULSK LMC ML | 2023 | This work |
| SM65 | Clinical | VULSK LMC ML | 2023 | This work |
| SM66 | Clinical | VULSK LMC ML | 2023 | This work |
| SM67 | Clinical | VULSK LMC ML | 2023 | This work |
| SM68 | Clinical | VULSK LMC ML | 2023 | This work |
| SM69 | Clinical | VULSK LMC ML | 2023 | This work |
| SM70 | Clinical | VULSK LMC ML | 2023 | This work |
| SM71 | Clinical | VULSK LMC ML | 2023 | This work |
| SM72 | Clinical | VULSK LMC ML | 2023 | This work |
| SM73 | Clinical | VULSK LMC ML | 2023 | This work |
| SM74 | Clinical | VULSK LMC ML | 2023 | This work |
| SM75 | Clinical | VULSK LMC ML | 2023 | This work |
| SM76 | Clinical | VULSK LMC ML | 2023 | This work |
| SM77 | Clinical | VULSK LMC ML | 2023 | This work |
| SM78 | Clinical | VULSK LMC ML | 2023 | This work |
| SM79 | Clinical | VULSK LMC ML | 2023 | This work |
| SM80 | Clinical | VULSK LMC ML | 2023 | This work |
| SM81 | Clinical | VULSK LMC ML | 2023 | This work |
| SM82 | Clinical | VULSK LMC ML | 2023 | This work |
| SM83 | Clinical | VULSK LMC ML | 2023 | This work |
| SM84 | Clinical | VULSK LMC ML | 2023 | This work |
| SM85 | Clinical | VULSK LMC ML | 2023 | This work |
| SM86 | Clinical | VULSK LMC ML | 2023 | This work |
| SM87 | Clinical | VULSK LMC ML | 2023 | This work |
| SM88 | Clinical | VULSK LMC ML | 2023 | This work |
| SM89 | Clinical | VULSK LMC ML | 2023 | This work |
| SM90 | Clinical | VULSK LMC ML | 2023 | This work |
| SM91 | Clinical | VULSK LMC ML | 2023 | This work |
| SM92 | Clinical | VULSK LMC ML | 2023 | This work |
| SM93 | Clinical | VULSK LMC ML | 2023 | This work |
| SM94 | Clinical | VULSK LMC ML | 2023 | This work |
| SM95 | Clinical | VULSK LMC ML | 2023 | This work |
| SM96 | Clinical | VULSK LMC ML | 2023 | This work |
| SM97 | Clinical | VULSK LMC ML | 2023 | This work |
| SM98 | Clinical | VULSK LMC ML | 2023 | This work |
| SM99 | Clinical | VULSK LMC ML | 2023 | This work |
| SM101 | Clinical | VULSK LMC ML | 2023 | This work |
| SM102 | Clinical | VULSK LMC ML | 2023 | This work |
| SM103 | Clinical | VULSK LMC ML | 2023 | This work |
| SM104 | Clinical | VULSK LMC ML | 2023 | This work |
| SM105 | Clinical | VULSK LMC ML | 2023 | This work |
| SM106 | Clinical | VULSK LMC ML | 2023 | This work |
| SM107 | Clinical | VULSK LMC ML | 2023 | This work |
| SM108 | Clinical | VULSK LMC ML | 2023 | This work |
| SM109 | Clinical | VULSK LMC ML | 2023 | This work |
| SM110 | Clinical | VULSK LMC ML | 2023 | This work |

*Environmental *S. maltophilia* was isolated from soil (samples collected in various locations of Lithuania) and fish gut; clinical isolates were collected from infected patients from several healthcare centers in Lithuania: NCI – National Cancer Institute (Lithuania); VCCH LM –Vilnius City Clinical Hospital, Laboratory of Microbiology; VUH SK PD – Vilnius University Hospital Santaros Clinics, Pediatrics Department, Division of Infectious Diseases; KCPHC – Kaunas City Public Health Center, VULSK LMC ML – Vilnius University Hospital Santaros Clinics, Center of Laboratory Medicine, Laboratory of Microbiology.

**Supplementary Table 5.** Oligonucleotides used in this study.

| **Primer name** | **Primer sequence 5' – 3'** | **Primer function** | **Reference** |
| --- | --- | --- | --- |
| pUC19_cat | TCTCTAGAGGATCCCCTTAATGAATCGGCCAACGCG | Chloramphenicol resistance gene amplification (for mutagenesis ) | This work |
| N_cat_F | GCCCTGGGCCAACTTTTGG |  |  |
| pUC19_wzc_5_F | AATTCGAGCTCGGTACCCAGAGTTCTGGATGCTCAAGTTCCG | Amplification of upstream region of CPS operon in SM21 |  |
| wzc_5_R | CGTTCCGGCAGTTACTCGGTCACATACAGGTGCGGGCAGCAA |  |  |
| membr_3_F | GGAACGAGGTGGTGTGTGAGC | Amplification downstream region of CPS operon in SM21 |  |
| membr_3_R | CCAAAAGTTGGCCCAGGGCCCTTCAAGCTCGTCAAGCAGC |  |  |
| wzc_check_F | CGGCGTGAAGCTGAGTTCGC | Verification of capsule operon deletion in SM21 |  |
| membr_check_R | CAGGTAAATGCCTCCCGGTGG |  |  |
| wzc48_3_R | CCAAAAGTTGGCCCAGGGCAATCAGTGAGCAGAGTGGGTGC | Amplification of the *wzc* coding gene upstream region in SM48 |  |
| wzc48_3_F | CCTGCCCTGCGAAGTGGTCAGACTCCTCATAGCTTCCACTTCCGT |  |  |
| wzc48_5_R | TGACCACTTCGCAGGGCAGG | Amplification of *wzc* coding gene downstream region in SM48 |  |
| wzc48_5_F | GCCAGTGAATTCGAGCTCGGTACCCCTTCTCAATGCAGCGCCAGG |  |  |
| wzc48_check_F | TACCCTCCCAGGTTCGATGGC | Verification of *wzc* gene deletion in SM48 |  |
| wzc48_check_R | TGACGCCTGCAATGATCTGTCG |  |  |
| MuacF | TGTCCACCTTCCAGCAGATGT | Evaluation of mouse actin coding gene expression | (5) |
| MuacR | TCAGTAACAGTCCGCCT |  |  |
| MutnF | AGGAGAAAGTCAACCTCCT | Evaluation of mouse *Tnf* gene expression | (5) |
| MutnR | AAAGTAGACCTGCCCGGAC |  |  |
| Mu6F | TCCAGTTGCCTTCTTGGGAC | Evaluation of mouse *Il6* gene expression | (5) |
| Mu6R | GTACTCCAGAAGACCAGAGG |  |  |
| mNLRP3 RT fwd | CGAGACCTCTGGGAAAAAGCT | Evaluation of mouse *Nlrp3* gene expression | (5) |
| mNLRP3 RT rev | GCATACCATAGAGGAATGTGATGTACA |  |  |
| mCaspase-1 RT fwd | TTTCAGTAGCTCTGCGGTGT | Evaluation of mouse *Casp1* gene expression | (5) |
| mCaspase-1 RT rev | TTTCTTCCTGATTCAGCACTCTC |  |  |
| mCaspase-11 RT fwd | GCCACTTGCCAGGTCTACGAG | Evaluation of mouse *Casp11* gene expression | (5) |
| mCaspase-11 RT rev | AGGCCTGCACAATGATGACTTT |  |  |
| IL18F | AGGACACTTTCTTGCTTGCC | Evaluation of mouse *Il18* gene expression | (5) |
| IL18R | CACAAACCCTCCCCACCTAA |  |  |
| IL-betaF | TGGACCTTCCAGGATGAGGACA | Evaluation of mouse *Il1b* gene expression | (5) |
| IL-betaR | GTTCATCTCGGAGCCTGTAGTG |  |  |
| IL-10R | CGGTTAGCAGTATGTTGTCCAGC | Evaluation of mouse *Il10* gene expression | (6) |
| IL-10F | CGGGAAGACAATAACTGCACCC |  |  |
| MIP-2F | CATCCAGAGCTTGAGTGTGACG | Evaluation of mouse *Cxcl2* gene expression | (7) |
| MIP-2R | GGCTTCAGGGTCAAGGCAAACT |  |  |

**References**

1.	Klimkaitė L, Armalytė J, Skerniškytė J, Sužiedėlienė E. The Toxin-Antitoxin Systems of the Opportunistic Pathogen Stenotrophomonas maltophilia of Environmental and Clinical Origin. Toxins (Basel). 2020 Oct 1;12(10):635.

2.	Klimkaitė L, Drevinskaitė ,Radvilė, Krinickis ,Karolis, Sužiedėlienė ,Edita, and Armalytė J. Stenotrophomonas maltophilia of clinical origin display higher temperature tolerance comparing with environmental isolates. Virulence. 2025 Dec 31;16(1):2498669.

3.	Lira F, Berg G, Martínez JL. Double-Face Meets the Bacterial World: The Opportunistic Pathogen Stenotrophomonas maltophilia. Frontiers in Microbiology [Internet]. 2017 [cited 2024 Jan 20];8. Available from: https://www.frontiersin.org/articles/10.3389/fmicb.2017.02190

4.	Lira F, Hernández A, Belda E, Sánchez MB, Moya A, Silva FJ, et al. Whole-Genome Sequence of Stenotrophomonas maltophilia D457, a Clinical Isolate and a Model Strain. J Bacteriol. 2012 Jul;194(13):3563–4.

5.	Skerniškytė J, Karazijaitė E, Lučiūnaitė A, Sužiedėlienė E. OmpA Protein-Deficient Acinetobacter baumannii Outer Membrane Vesicles Trigger Reduced Inflammatory Response. Pathogens. 2021 Apr;10(4):407.

6.	Tian Y, Wang J, Wang W, Ding Y, Sun Z, Zhang Q, et al. Mesenchymal stem cells improve mouse non-heart-beating liver graft survival by inhibiting Kupffer cell apoptosis via TLR4-ERK1/2-Fas/FasL-caspase3 pathway regulation. Stem Cell Res Ther. 2016 Oct 27;7(1):157.

7.	Saup R, Nair N, Shen J, Schmaus A, Thiele W, Garvalov BK, et al. Increased Circulating Osteopontin Levels Promote Primary Tumour Growth, but Do Not Induce Metastasis in Melanoma. Biomedicines. 2023 Apr;11(4):1038.
